# Subgroup specific transcriptional regulation of salmonid non-classical MHC class I L lineage genes following viral challenges and interferon stimulations

**DOI:** 10.3389/fimmu.2024.1463345

**Published:** 2024-12-20

**Authors:** Maryam Imam, Atefeh Kianian, Shripathi Bhat, Viktoria Erika Fure Lukes, Linn Greiner-Tollersrud, Eva-Stina Edholm

**Affiliations:** Norwegian College of Fishery Science, Faculty of Bioscience, Fisheries and Economics, University of Tromsø – The Arctic University of Norway, Tromsø, Norway

**Keywords:** MHC, non-classical MHC class I, L lineage, Atlantic salmon (Salmo salar L.), Transcriptional (regulation)

## Abstract

Non-classical MHC class I genes which, compared to classical MHC class I, are typically less polymorphic and have more restricted expression patterns are attracting interest because of their potential to regulate immune responses to various pathogens. In salmonids, among the numerous non-classical MHC class I genes identified to date, L lineage genes, including Sasa-*LIA* and *Sasa-LGA1*, are differentially induced in response to microbial challenges. In the present study, we show that while transcription of both *Sasa-LIA* and *Sasa-LGA1* are induced in response to SAV3 infection the transcriptional induction patterns are distinct for each gene. While elevated *Sasa-LGA1* expression is maintained long-term following *in vivo* SAV3 infection *Sasa-LIA* expression is transient, returning to near baseline weeks prior to viral clearance. Furthermore, by contrasting L lineage transcriptional induction potential of SAV3 with that of IPNV we show that *Sasa-LIA* and *Sasa-LGA1* transcriptional induction is tightly interconnected with select type I and type II interferon induction. Both type I and type II interferon stimulation, to varying degrees, induce *Sasa-LIA* and *Sasa-LGA1* expression. Compared to IFNa1 and IFNc, IFN-gamma was a more effective inducer of both *Sasa-LIA* and *Sasa-LGA1* while IFNb showed no activity. Furthermore, IFNa was a more potent inducer of Sasa-LIA compared to IFNc. The involvement of type I IFN and IFN gamma in regulation of *Sasa-LIA* and *Sasa-LGA1* expression was further substantiated by analysis of their respective promoter regions which indicate that ISRE and GAS like elements most likely cooperatively regulate *Sasa-LIA* expression while IFN gamma induced expression of *Sasa-LGA1* is critically dependent on a single, proximally located ISRE element. Together, these findings imply that Sasa-*LIA* and *Sasa-LGA1* play important but likely functionally distinct roles in the anti-viral response of salmonids and that these two molecules may serve as immune regulators promoting more effective antiviral states.

## 1 Introduction

Non-classical MHC class I genes encode for a heterogeneous group of molecules that in mammals have been shown to possess diverse functional roles (reviewed in (1)). Unlike classical MHC class I molecules which are expressed on the surface of most cells, many non-classical MHC class I genes have more restricted expression patterns (2, 3). Common to many non-classical MHC class I genes is the lack of the canonical S-X-Y promoter module which, when cooperatively bound by a multiplex protein complex, consisting of several transcription factors bound to NLRC5, drive transactivation of classical MHC class I genes in mammals (4, 5). Thus, differing from classical MHC class I genes, distinct transcriptional and post-transcriptional control mechanisms are involved in regulating basal and inducible expression of many non-classical MHC class I genes (6–11).

In salmonids, non-classical MHC class I L lineage genes are encoded outside the classical MHC class I core region and are found scattered across multiple chromosomes (12). Although salmonid L lineage genes share structural similarities with their classical MHC class I counterparts, the overall sequence identity is low and common to all L lineage sequences is a lack of canonical peptide anchoring residues and a relatively high hydrophobicity within the antigen binding groove (13, 14). In Atlantic salmon, low to modest constitutive expression of L lineage genes is evident in primary and secondary lymphoid tissues as well as in mucosal organs with some genes, such as *Sasa-LFA*, displaying a highly tissue restricted expression pattern (15). However, while basal L lineage mRNA expression is markedly lower, ranging between 50 to 100-fold lower compared to that of classical MHC class I (14, 15), during pathological conditions, such as viral and to a lesser extent bacterial infections, discrete L lineage gene transcription is induced (15). For example, intraperitoneal infection of Atlantic salmon parr with the salmonid alpha virus 3 (SAV3), an enveloped, single-stranded, positive-sense RNA virus, elicits induction of distinct L lineage genes implying that, *Sasa-LIA* but also, *Sasa-LGA1* and to a lesser extent *Sasa-LHA* are involved in the early anti-viral immune response of Atlantic salmon (15). In support of this, type I IFNa1 induces *Sasa-LIA* and *Sasa-LGA1* mRNA levels in Atlantic salmon kidney (ASK) cells (15). Moreover, IFNγ stimulation moderately modifies *Sasa-LIA* and *Sasa-LGA1* gene expression in Atlantic salmon head kidney (SHK 1) cells (16) hinting at regulatory roles of both type I and type II IFNs in L lineage gene induction.

In mammals, it is well documented that type I IFNs induce expression of classical MHC class I genes and that a major component of the antiviral properties of type I IFNs is related to the interferon induced regulation of genes involved in processing and presentation of antigens (17–19). Type I interferon induced MHC class I transcription is mediated through both canonical and non-canonical JAK–STAT signaling pathways involving STAT1-STAT2 heterodimers and STAT1 homodimers and is dependent on IFN regulatory factors (IRFs), that bind to interferon stimulated response elements (ISRE) within the MHC class I promoters (20, 21). Similarly, IFNγ enhance expression of MHC class I through induction of IRF1, which in turn bind to ISRE elements in the MHC class I promoter (18). In fish, like in mammals, classical MHC class I is ubiquitously expressed and transcription is upregulated in response to viral infections (22, 23) and interferon stimulations (16). The promoter region of classical MHC class I in rainbow trout and Atlantic salmon, like that of higher vertebrates, contains an S-X-Y-like motif as well as a conserved ISRE element and multiple putative Gamma interferon activation (GAS-) and IRF sites, supporting IFN type I and type II -inducible properties (22, 24). Comparably, the promoter region of Atlantic salmon non-classical L lineage genes contains subgroup specific regulatory elements, which are potentially targeted by IFN regulatory factors (IRFs) and signal transducer and activator of transcription (STAT) proteins but lack a S-X-Y motif (15). These results are indicative of a large functional diversity among salmonid L lineage genes raising basic questions about how the expressions of these genes are regulated.

To date, type I IFN and type II IFN genes have been cloned and sequenced in several fish species and both types of IFNs have been produced as recombinant proteins, which have been shown to possess distinct biological activities (25–27). Atlantic salmon possess a broad repertoire of type I IFN genes encompassing at least 6 different classes (IFNa-IFNf) that may be classified into two groups based on the presence of two or four cysteines, forming one or two disulphide bonds, respectively (28). Among these, the 2-cysteine containing IFNa and the 4-cysteine containing IFNb and IFNc all induce expression of antiviral proteins and various transcription factors, including IRFs, albeit with different potentials (26). Comparably, fish IFNγ has been shown to induce expression of classical MHC class II (29) and classical MHC class I as well as a large number of genes encoding for proteins related to antigen processing and presentation (16). Like type I IFNs, rIFNγ also stimulates gene expression of the antiviral proteins Mx, suggesting some cross talk among the IFN signaling pathways (30).

While fish and mammalian IFNs have evolved quite differently and are not orthologs they appear to mediate their cellular responses in a similar manner utilizing cell specific receptors and activation of signaling pathways, predominantly dependent on sequential phosphorylation of the tyrosine residues of the Janus tyrosine kinases/signal transducers and activators of transcription proteins (JAK/STAT) which modulates the expression of specific genes and enhance antiviral mechanisms (reviewed in (31, 32)). Several Atlantic salmon type I IFN receptors, which likely show selectivity in binding affinity to different IFNs, have been identified, allowing for a large potential functional variation among the type I IFNs (33).

In this study, we undertook a comprehensive analysis detailing viral and interferon induced transcriptional induction patterns of Atlantic salmon L lineage genes, focusing on *Sasa*-*LIA* and Sasa-*LGA1*. Expression kinetics of L lineage genes were assessed both *in vitro* and *in vivo* following viral challenges, demonstrating that while both Sasa-*LIA* and Sasa-*LGA1* are upregulated in response to SAV3 but not infectious pancreatic necrosis virus (IPNV) infection, there is a clear difference in kinetics and temporal regulation between these two genes indicating a tight regulatory control and functional diversification. Furthermore, characterization of type I and type II interferon induced transcription of Sasa-*LIA* and Sasa-*LGA1* revealed specific induction patterns that are regulated by subgroup specific, evolutionary conserved promoter elements.

## 2 Materials and methods

### 2.1 Experimental animals and infection protocols

Samples from SAV3-infected Atlantic salmon were obtained from an *in vivo* SAV3 challenge trial described in detail elsewhere (34). Briefly, rSAV3 (referring to SAV3 virus recovered from cells transfected with a plasmid containing the entire SAV3 genome (prSAV3; originally cloned from the wild-type SAV3 isolate H20/03 (DQ149204, AY604236)) was used. The same prSAV3 plasmid was used as a backbone for the construction of SAV3 infectious strains with targeted mutations, two of which (SAV3-E2_319A_ and rSAV3-Cap_NLS_) were included in this study. SAV3-E2_319A_ has a mutation in the predicted N-linked glycosylation motif in the E2 protein while rSAV3-CapNLS has a mutation in the subcellular localization signals of the capsid protein (35). Both attenuated SAV3 strains are infectious, transmittable to naïve cohabitant fish and induce innate anti-viral responses (34).

The *in vivo* experiments were conducted at the Aquaculture Research Station, Tromsø, Norway. The challenge trials were approved by the Norwegian Animal Research Authority (NFDA) according to the European Union Directive 2010/63/EU (permit numbers 16409 and 19014) and were performed in accordance with the recommendations of the current animal welfare regulations: FOR-1996-01-15-23 (Norway). Briefly, non-vaccinated Atlantic salmon, pre-smolts, strain NLA, were reared in a hatchery at the Aquaculture Research Station, Tromsø, Norway and confirmed free of the salmon pathogens ISAV, SAV, PRV and IPNV by RT-RT-qPCR. Fish were kept in running freshwater at 10°C, exposed to continuous light and fed with commercial dry feed and starved for 24 hours prior to handling and sampling. The fish were randomly selected for immunization, anesthetized by bath immersion in benzocaine chloride (0.5 g/10 L) for 2–5 min, labelled (tattoo) and intramuscularly (i.m.) injected. In this study, samples from control fish i.m. injected with PBS and fish i.m. infected with 0.2 ml cell culture medium containing 10^2^ TCID50 virus [rSAV3, rSAV3-E2_319A_ or rSAV3-Cap_NLS_ (34)] or with a vaccine based on inactivated SAV3 with water-in-oil adjuvant (36) (kindly supplied by PHARMAQ AS) were analyzed. Shedder fish injected with SAV3 H20/03/2 at week 7 [as described in (34)], were added after sampling at week 8. Samples from 6-fish/time point were collected through a 12-week sampling period. Organs (heart, spleen, head kidney and gill) from virus-challenged and control fish were aseptically collected at 2, 4, 6-, 8-, 10- and 12-weeks post infection and kept in RNA-later. The organs were used for gene expression analyses by RT-qPCR following RNA isolation and subsequent cDNA synthesis as described below.

### 2.2 Cell lines and virus

SSP-9 cells derived from Atlantic salmon head kidney (HK) (37), were kindly provided by Dr. S. Perez-Prieto (CSIC, Madrid, Spain). Chinook salmon embryo (CHSE-214) cells were purchased from American Type Culture Collection. Cell lines were maintained as monolayers in Leibovitz’s medium with L-glutamine (L-15) (Life Technologies) supplemented with antibiotics (10 U/ml penicillin, 10 μg/ml streptomycin) and 8% fetal bovine serum (FBS) at 20°C. For all *in vitro* infection trials Salmonid alphavirus subtype 3 (SAV3) (PDV-H10-PA3, provided by Professor Øystein Evensen, Norwegian University of Life Sciences) was used. The SAV3 virus was propagated in CHH-1 cells in L-15+ with 5% FBS at 15°C. Virus titer was determined by the TCID_50_ method. Infectious pancreatic virus (IPNV) [strain N1, serotype Sp (38)] were propagation in CHSE-214 cells and titrated as described in (39).

### 2.3 *In vitro* SAV3 and IPNV infection

SSP9 and CHSE-214 cells were seeded in 6-well plates at a density of approx. 2,5 × 10^5^ cells/ml and grown until 70% confluency. Culture media was removed, and cells were washed two times with 2 ml sterile PBS followed by addition of serum and antibiotic free medium containing SAV3 or IPNV (MOI = 0,1, MOI= 1 or MOI= 5). After allowing the virus to be absorbed for 3 h, the medium was replaced with L-15+ supplemented with 2% FBS and cells were incubated for 1, 3, 5, 7, 9, 10 and 12 days following SAV3 challenge and 6, 12, 24, 48 and 72 h following IPNV infection. Variable time points for RNA isolation were based on documented differences in SAV3 and IPNV replication kinetics in CHSE-214 cells wherein in for IPNV, viral specific RNA synthesis peaks 8-10 hours after infection (40) typically resulting in significant (>70%) CPE by 48 hours post infection rendering expression analysis at later time points challenging and largely uninformative (41, 42). In contrast, SAV3 infection of CHSE typically result in detectable viral transcripts around 2 dpi with minor CPE observed at 6 dpi (41, 42). Following respective incubation time points, RNA was isolated for cDNA synthesis and RT-qPCR (described in detail below).

### 2.4 Reagents

Recombinant Atlantic salmon IFNa1 (GenBank accession no. DQ354152.1), IFNc (GenBank accession no. JX524153) and IFNb (GeneBank accession no. JX524152) were produced by transfection of sub confluent HEK-293 cells with IFN expression plasmids as previously described (25). Rainbow trout (*Oncorhynchus mykiss*) IFNγ was produced in E. coli (43) and protein concentrations were measured with a QuickStart Bradford protein assay kit (Bio-Rad) with bovine serum albumin as a standard. JAK I inhibitor was obtained from Calbiochem (CAS 457081-03-7).

### 2.5 Primary Head kidney cell isolation and stimulation

Total leukocyte populations from Head-kidney (HK) were isolated on Percoll (GE Healthcare) gradients following previously established protocols (44). Briefly, HK were sampled aseptically and kept in ice-cold transport medium (L-15 medium with 10 U/ml penicillin, 10 μg/ml streptomycin, 2% fetal bovine serum, 20 U/ml heparin) until homogenization on 100 μm cells strainers (Falcon). The resulting cell suspensions were layered on 25/54% discontinuous Percoll gradients and centrifuged at 400 × *g* for 40 min at 4°C. Cells at the interface were collected and washed twice in L-15 medium. Cells were counted using an automatic cell counter (Countess II Automated cell counter, Thermo Fisher, cat. nr. AMQAF1000). A total of 3 x 10^6^ cells/ml were seeded in 1 ml L-15+ media supplemented with 8% FBS in 24 well plates (Nunclon Delta Surface, Thermo Scientific), stimulated with 500U of rIFNa, rIFNb, rIFNc or 10ng/ml IFNγ and cultivated at 16°C for 6, 12, 24 or 48 hours. Cells cultured in L15+/8% FBS media alone and harvested at the corresponding time points were used as controls. For inhibitory studies HKLs were isolated and stimulated as described above and incubated for 12 hours in the absence or presence of 15 nmol/ml of JAK I inhibitor, cells with inhibitor alone were included as controls. Following respective incubation time points, RNA was isolated for cDNA synthesis and RT-qPCR (described in detail below).

### 2.6 SSP9 and CHSE stimulations

SSP-9 cells (7× 10^5^ cells/well) and CHSE cells (7 x 10^5^ cells/well) were seeded in 1 ml culture media in 6 well culture plates. Cells in triplicate wells were stimulated with 1000 U/ml rIFNa, 1000 U/ml rIFNc or 10 ng/ml rIFNγ and harvested at different time points. For inhibitory studies SSP9 cells were stimulated as described above and incubated for 12 hours in the absence or presence of 15 nmol or 150 nmol of JAK I inhibitor, cells with inhibitor alone were included as controls. Following respective incubation time points, RNA was isolated for cDNA synthesis and RT-qPCR (described in detail below).

### 2.7 Western blot

SSP-9 cells grown in 6 well plates as described above were stimulated with 10 ng/ml rIFNγ in the presence or absence of JAK I inhibitor were harvested at 12 hps, washed and lysed in M-PER (Mammalian protein extraction buffer) supplemented with HALT Protease inhibitor (Thermo fisher) and subjected to SDS-PAGE using NuPAGE Novex Bis-Tris 4–12% gels (Life Technologies). Proteins were transferred to PVDF membrane and incubated in blocking agent (Intercept Blocking Buffer, Licor) in TBS-T overnight at 4°. The membranes were incubated with either a polyclonal rabbit antibody prepared against Atlantic salmon Mx1 diluted 1/3000 (v/v) followed by an incubation with goat anti-rabbit Ig (H+L) secondary antibody conjugated to Alexa fluor 680 diluted 1/10000 (v/v) for 1 h at room temperature or actin 1/1000 (v/v) followed by incubation with a goat-anti-mouse Ig (H+L) secondary antibody conjugated to Alexa fluor 680 diluted 1/10000 (v/v) for 1 h at room temperature. After washing the membrane was imaged using Odyssey CLX infrared imaging system.

### 2.8 Microscopy

To study the temporal progression of IPNV and SAV3 infections (up to 72 hours and five days, respectively) along with control (uninfected CHSE-214), 2,25 × 10^5^ CHSE-214 cells were seeded in 35mm confocal dishes and allowed to adhere overnight. Culture media was removed, and cells were washed two times with 2 ml sterile PBS followed by addition of serum- and antibiotic free medium containing SAV3 or IPNV (MOI= 1). After allowing the virus to be absorbed for 3 h, the medium was replaced with L-15+ supplemented with 2% FBS and cells were incubated for 1, 3, 5 and 7 days following SAV3 challenge and 6, 12, 24 and 48 h following IPNV infection. Following respective incubation time points, cells were fixed. For fixation, PFA was used for twenty minutes followed by three washes with PBS. The cell membranes were stained with CellMask™ orange followed by NucSpot^®^ Live direct dye for nuclear staining and stored until imaging. Images were acquired using a DeltaVision OMX V4 Blaze imaging system from GE Healthcare Life Sciences equipped with a 60X 1.42NA oil-immersion objective from Olympus; three sCMOS cameras; and lasers for excitation at wavelengths of 488 nm, 568 nm, and 642 nm. The exposure time and illumination power were adjusted to obtain a maximum of 10000 grayscale counts on the camera chip. A total of ten z-plane frames with z-steps of 100nm were collected on each imaging channel along the optical axis of the objective. The frames were automatically stitched into an 8×8 tile mosaic image by the built-in software package SoftWoRx.

### 2.9 Quantitative PCR and transcript analysis

Total RNA from tissues, primary leucocytes, SSP9 and CHSE cell lines were isolated using the RNeasy Mini Kit (Qiagen) following the manufacturer`s recommendation and RNA was quantified using NanoDrop (ND 1000 Spectrophotometer). For RNA isolated from tissues and primary leucocytes 1000 and 500 ng respectively was treated using DNase I to remove all residual genomic DNA. Twenty microliter cDNA reactions were synthesized using TacMan reverse transcription reagents (Applied Biosystems) using random hexamer primers under the following conditions: 25°C for 10 min, 37°C for 30 min and 95°C for 5 min. cDNA samples were diluted 1:2 and stored at -20°C until use. For SSP9 and CHSE cells cDNA was synthesized using the QuantiTect RT kit (Qiagen) according to the manufacturer`s instructions with 500ng of RNA per 20 µl of reaction. cDNA was diluted 1:2 and stored at -20°C until use. Quantitative PCR (RT-qPCR) was run as 10ul duplicate reactions on a 7500 Fast Real-Time PCR systems (Applied Biosystems) according to standard protocol. All primers were validated prior to use and primer sequences are available upon request. For each primer pair and tissue/cell a negative control (no template) and a no reverse transcriptase control RT (-) was performed. A threshold difference of at least 6 quantification cycles (Cq) between Rt (+) and RT (-) was used as a cut-off. Ct values >38 was rejected. Parameters were as follows: 2 min 95°C followed by 40 cycles of 95°C for 15 seconds and 60°C for 1 min. Melt curve analysis were performed to ensure that there were no artifacts, and a single product was amplified. Relative quantitative PCR gene expression analysis was performed using the ΔΔCt method. Expression of individual genes was examined relative to the endogenous EF1-α controlled. Relative expression (zero-hour samples) was calculated using the 2-ΔCt method. For infected tissues and stimulated cells, fold change or alternatively log2 fold change was calculated against the appropriate controls.

### 2.10 Plasmid construct design

*Sasa-LGA1* and *Sasa-LIA* promoter regions containing 2000 bp upstream and 75 bp downstream of the transcriptional start site were extracted from the Salmo salar genome NC_027320, GCF_000233375.1. First, the transcription factor biding sites were identified using the online application PROMO which uses TRANSFAC database v8.3.0 and constructs positional weight matrices in a species or taxon using known transcription factor binding sites and then search for matches in query DNA sequences (45). Based on these results. The 2000 bp sequence was further processed to create different constructs for luciferase assay containing different promoters. All constructs were synthesized in PGL basic promoter by Twist Biosciences, San Francisco. The full 2000 bp upstream promoter was synthesized in PGL basic promoter for both Sasa-LGA and Sasa-LIA. For Sasa-LIA truncated promoter construct were designed to either including or exclude specific ISRE and GAS elements; -150/+75 including the ISRE and GAS elements, along with -700/+75 bp, -1200/+75 bp, -1200/-700 bp (including +75 at 5’). The mutated constructs were focused on only ISRE and GAS elements. The Sasa-LIA-150 + 75 bp construct was selected to mutate ISRE element GAAAGTGAAA to CCGAGTGACG and GAS- like element TTCAGAA to GCGAGCG. Similarly, for Sasa-LGA a systematic deletion of promoter was done with +75-200 including the IRF1/3 and ISRE element, +75-500 including STAT binding site, -1200/+75, and -1300/-1900 (including +75 at TSS) that included putative GAS like element but no proximal ISRE elements. For mutation, the ISRE element in the -200/+75 construct was mutated.

### 2.11 Luciferase assay

CHSE-214 were seeded into 96-well culture plates at a density of 1.6 × 10^4^ cells/well in 100ul of L-15 medium supplemented with 8% FBS and grown to 50% confluence overnight at 20 °C. The cells were transiently transfected by adding a transfection mix consisting of 10 µl Opti-MEM^®^ (Life Technologies) solution containing 90 ng of promoter reporter (firefly luciferase) construct, 10 ng Renilla luciferase vector (Promega- Madison WI), and 0.3 µl TransIT-LT1 per well. Atlantic salmon LIA and LGA1 promoter constructs were investigated, while pGL3-basic was included as empty vector control. All promoter fragments and mutants were synthesized by Twist bioscience and cloned into pGL3-basic. 24 hours post transfection, cells were stimulated with 600 U/ml of recombinant (r)Type I rIFNa or rIFNc or 30 ng/ml of rINFγ. Luciferase activity was measured 48 h post stimulation using the Dual-Luciferase^®^ Reporter Assay System (Promega, Madison, WI) according to the manufacturer’s protocol. The constitutively expressing Renilla luciferase construct provided an internal control value to which the expression of the experimental firefly luciferase was normalized. The firefly and Renilla luciferase activity was measured in a Luminoscan RT luminometer, in which all samples for the luciferase assay were set up in four parallels for each treatment and the results are given as relative light units (RLU). The results are presented as fold change in relative light units (RLU) by dividing the RLU of the stimulated samples by the average RLU of the corresponding non-stimulated samples.

### 2.12 Statistical analysis

For *in vivo* studies all quantitative data were based on duplicated measurements from a minimum of four fish n ϵ (46) and were analyzed in GraphPad Prism 8. For primary cells and cell lines all quantitative data were based on triplicated samples undergoing duplicated measurements. All *in vitro* experiments were repeated a minimum of three times in independent experiments. Statistical evaluations were performed using Tukey`s multiple comparisons test following a significant one-way ANOVA. Correlation among MHC class I L lineage expression, pathogen load and interferon expression were determined using the Pearson Correlation coefficiency (p=0.05) calculated from the relative expression of each gene normalized to EF1-alpha(B). For all analysis a p value < 0.05 was considered significant.

### 2.13 Data mining and phylogenetic analysis

Genome searches were performed using previously identified Atlantic salmon MHC class I L lineage LIA and LGA1 sequences and blasted against annotated salmonid genomes available in NCBI using mega blast. Genomic regions identified through these searches were screened for LIA and LGA1 leader sequences based on Atlantic salmon LIA and LGA1 cDNA sequences and the upstream proximal (-500) promoter regions were extracted. Genomes used in this study were as follows: *Oncorhyncus gorbuscha* GCA_021184085.1 (pink salmon), *Oncorhyncus keta* GCA_023373465.1 (chum salmon), *Oncorhynchus kisutch* GCA_002021735.2 (coho salmon), *Oncorhynchus mykiss* GCA_013265735.3 (rainbow trout), *Oncorhynchus nerka* GCA_006149115.2 (sockeye salmon), *Oncorhynchus tshawytscha* GCA_018296145.1 (Chinook salmon), *Salmo salar* GCA_905237065.2 (Atlantic salmon), *Salmo trutta*.

GCA_901001165.1 (brown trout), *Salvelinus* GCA_002910315.2 (unclassified species in the genus *Salvelinus)*, *Salvelinus fontinalis* GCA_029448725.1 (brook trout) and *Salvelinus namaycush* GCA_016432855.1 (lake trout). For promoter regions, all evolutionary analyses were conducted in MEGA7. Sequences were aligned using ClustalX with manual corrections for some predicted sequences and bootstrapped phylogenetic trees were constructed using the Maximum Likelihood method. The percentage of trees in which the associated taxa clustered together are shown next to the branches. The trees are drawn to scale, with branch lengths measured in the number of substitutions per site. Gene models were predicted based on expressed LIA and LGA1 sequences in Atlantic salmon.

## 3 Results

### 3.1 Non-classical MHC class I L lineage gene expression profiles following *in vivo* SAV3 challenge

**Figure 1:**
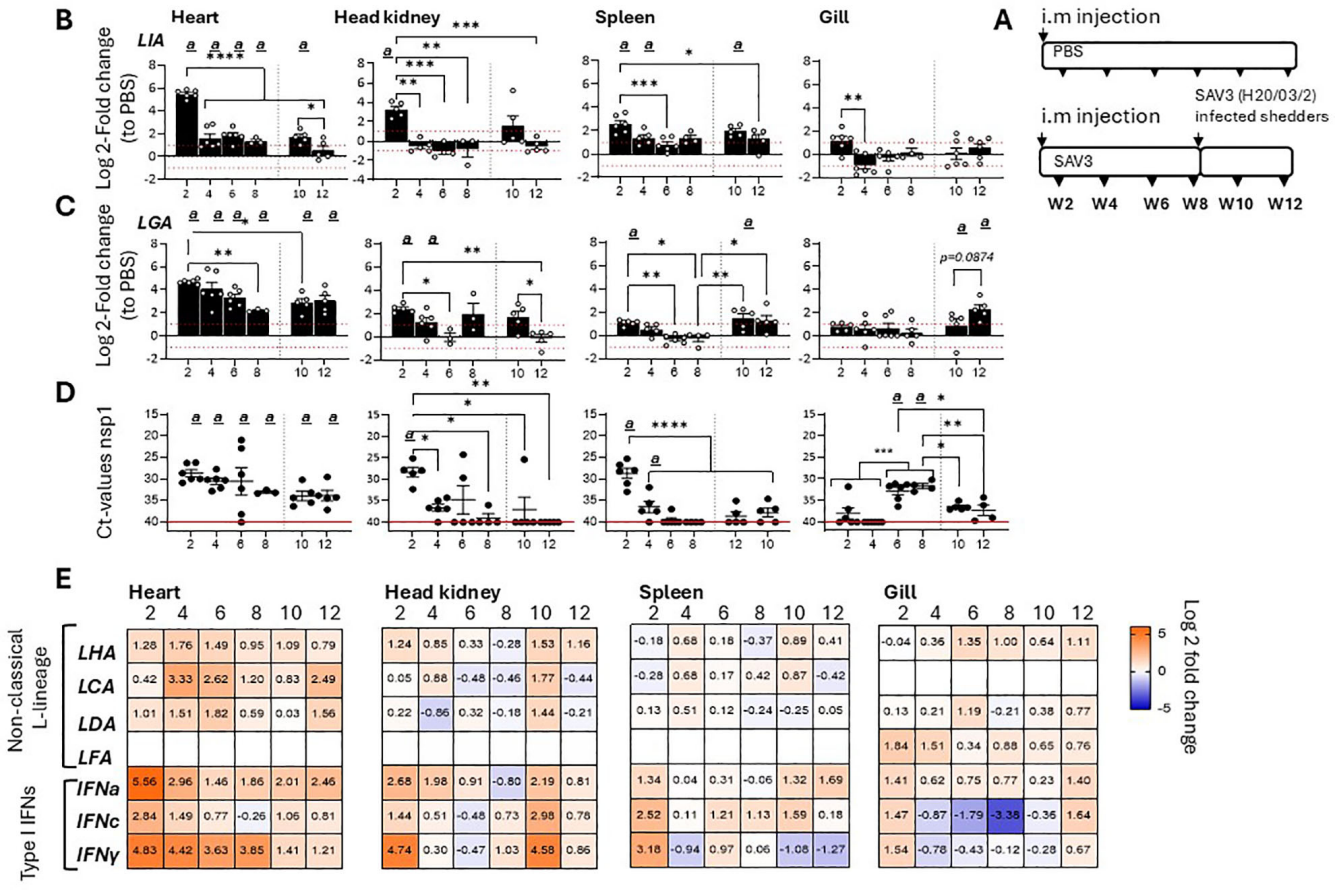
*Sasa-LIA* and Sasa-LGA1 are differentially regulated in response to SAV3 infection in vivo.**(A)** Schematic outline of the experimental setup indicating various sampling points and experimental groups. Log2 fold change in gene expression of L lineage MHC class I genes **(B)** *Sasa-LIA* and **(C)** Sasa-LGA1, normalized to the reference gene EF1α, in different tissues at various times post challenge (*n* ϵ 3-6), relative to the control groups from the same time point (*n* ϵ 6) is shown. Each dot represents an individual fish, the red dotted line intersecting the y-axis at 1 and -1 in **(B)** and **(C)** represent a 2-fold increase compared to PBS injected controls, significant *(p* < 0.05) upregulation compared to the corresponding control group is indicated by *(a)* and asterisks indicate the strength of significance among the different time points as indicated, *p < 0.05, **p < 0.01, ***p = 0.0001, ****p < 0.0001. **(D)** Individual Ct values and mean value of non-structural protein 1 (nsp1) detected by qPCR in heart, head-kidney spleen and gill at each sampling from 2- 12 wpi. **(E)** Heat maps illustrating average expression ratios of L lineage MHC class I genes (*Sasa-LHA, Sasa-LCA, Sasa-LDA* and *Sasa-LFA*), type I IFNa, type I IFNc and type II IFNγ as log2 fold-change values of the SAV3 infected groups relative to PBS injected controls for the indicated genes as measured by qPCR (bar graphs illustrating individual variation for each of the genes listed is shown in **Supplementary Figure 1**).

To further delineate gene specific SAV3 inducible responses among Atlantic salmon L lineage genes, tissue specific transcriptional induction and gene expression kinetics of six functionally expressed L lineage genes (*Sasa-LIA, Sasa-LGA1*, *Sasa-LHA, Sasa-LDA, Sasa-LFA* and *Sasa-LCA*) (15) were analyzed over a 12-week experimental immunization challenge using a recombinant SAV3 infectious strain. Accordingly, fish were i.m. infected with an infectious recombinant (r)SAV3 strain and subsequently challenged on week 8 by co-habitation with SAV3 (H20/03) infected shedder fish as previously described (34). At different times post-infection (2-, 4-, 6-, 8-, 10- and 12-weeks post infection [wpi]), log2 fold change in expression of the various L lineage genes in the heart (main site of viral replication), head-kidney, spleen and gill were determined (**Figure 1**; **Supplementary Figure 1**). Consistent with previous reports (15) *Sasa-LIA* and *Sasa-LGA1* gene expressions were markedly upregulated at the fist, 2 wpi, time point. For both *Sasa-LIA* and *Sasa-LGA1* the highest level of induction was observed in the heart, with an average fold increase of 46 (SD ± 10) and 25 (SD ± 3.3) respectively, followed by head-kidney and to a lesser extent spleen, with little to no transcriptional induction observed in the gill (**Figures 1B, C**). Notably, *Sasa-LIA* gene expression in the heart, despite the continuous detection of viral specific transcripts, was transient (**Figures 1B, C**). Across all examined tissues *Sasa-LIA* gene expression peaked at the 2-week time point followed by a return to near baseline levels by 4 wpi. In stark contrast, *Sasa-LGA1* expression in the heart remained elevated through the course of infection, albeit with a gradual decrease from 2 to 8 wpi. Similarly, 2 weeks after exposure to SAV3 infected shedders a moderate but transient induction of *Sasa-LIA* was observed in the heart and head-kidney with expression returning to baseline levels by the next sampling point. Comparably, post challenge *Sasa-LGA1* expression in the heart was significantly upregulated compared to PBS controls at both the 10- and 12-week time points. Neither *Sasa-LHA* nor *Sasa-LDA* were upregulated with a >2 log2-fold increase following SAV3 infection in any of the tissues examined with the exception of heart where significant upregulation of both genes was apparent at 4 wpi (**Figure 1E**, **Supplementary Figure 1**). For *Sasa-LCA* significant upregulation was observed in the heart at 4- and 6-wpi, which was reciprocated 4 weeks after addition of shedder fish (**Figure 1E**. **Supplementary Figure 1**). Consistent with previous results indicating a highly tissue specific expression pattern for some L lineage genes (15) *Sasa-LFA* expression was only detectable above threshold levels in the gills and showed no significant transcriptional response to SAV3 challenge. In the same sample set expression patterns of IFNa, IFNc and IFNγ were examined (**Figure 1E**, **Supplementary Figure 1**) revealing, as expected, a strong induction of type I IFNa, IFNc and type II IFNγ in response to SAV3 infection. Similar to what was observed for *Sasa-LIA*, IFNa expression in the heart peaked at 2 wpi, was significantly reduced by 4 wpi and continued to decline at the 6-, and 8 wpi time points. In contrast, elevated expression levels of both IFNc and IFNγ were maintained at the 2-, 4- and 6-wpi time points followed by a marginal reduction at 8 wpi.

**Figure 2:**
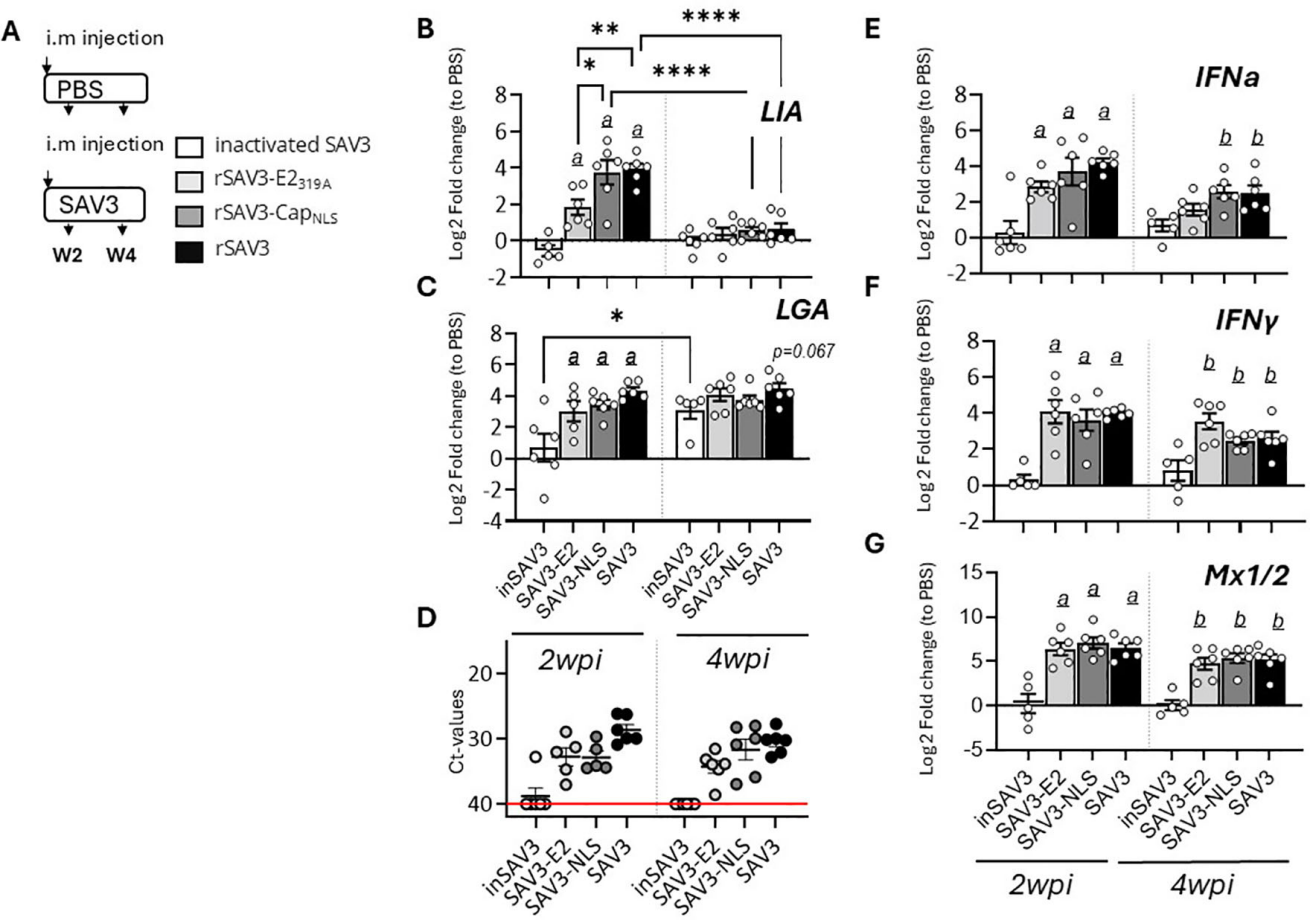
*Sasa-LIA* and Sasa-LGA1 expression in heart following infection with attenuated and inactivated SAV3. **(A)** Schematic outline of the experimental setup indicating various sampling points and experimental groups. **(B-C, E-G)** Log2 fold change in gene expression of *Sasa-LIA*, *Sasa-LGA1*, type I IFNa, type II IFNγ gamma and Mx1/2, normalized to the reference gene EF1α, at various times post challenge (*n* ϵ 6), relative to the control groups from the same time point (*n* ϵ 6) is shown. Asterisks indicate the strength of significance among the different time points as indicated, *p < 0.05, **p < 0.01, , ****p < 0.0001. Letters *(a* and *b)* above the bars in **(B-C**, **E-G)** indicate statistical significance compared to the group injected with inactivated SAV3 vaccine at 2- and 4-wpi respectively while *(c)* above the bars indicate statistical significance compared to the PBS injected group. **(D)** individual Ct value and mean value of nsp1 in in heart of groups infected with the different viral strains at 2- and 4wpi.

To investigate the factors governing discrete *Sasa-LIA* and *Sasa-LGA1* transcriptional induction patterns we examined the expression of these genes in fish i.m. infected with two genetically modified infectious strains of rSAV3 or with a vaccine based on inactivated SAV3 with water-in-oil adjuvants (36) (**Figure 2A**). These rSAV3 strains, which have previously been shown to actively infect and replicate in Atlantic salmon (34) are attenuated in either the envelope protein E2 (rSAV3-E2_319A_) or the capsid protein nuclear localization signal (rSAV3-Cap_NLS_). Similar to rSAV3, at 4 wpi both attenuated strains, as assessed by mRNA expression of select interferon stimulated genes, induce comparable anti-viral responses in the heart (34). Congruent with these observations, comparable levels of viral transcripts were detected in hearts isolated from fish infected with WT-rSAV3, rSAV3-E2_319A_ or rSAV3-Cap_NLS_ while no viral transcripts were detected in fish injected with the inactivated SAV3 (**Figure 2D**). While inactivated SAV3 failed to induce *Sasa-LIA* gene expression, significantly increased mRNA levels were detected at 2 wpi in response to WT-rSAV3, rSAV3-E2_319A_ and rSAV3-Cap_NLS_ (**Figure 2B**). However, *Sasa-LIA* expression in fish infected with rSAV3-E2_319A_ was significantly lower compared to both rSAV3-Cap_NLS_ and WT- rSAV3. As seen in **Figure 2C** *Sasa-LGA1* was upregulated in response to all three viral strains and, albeit not statistically significant, by 4 wpi inactivated SAV3 infection resulted in elevated *Sasa-LGA1* expression comparable to that of fish infected with actively replicating SAV3 strains. As a comparison no significant difference in expression of type I IFNa, type II IFN-y or the interferon inducible gene Mx1/2 were observed between rSAV3, rSAV3-E2_319A_ or rSAV3-Cap_NLS_ at 2 wpi (**Figures 2E-G**). However, at 4 wpi expression of IFNa in the rSAV3-E2_319A_ was lower compared to the other viral strains and did not reach significant induction levels compared to fish injected with inactivated SAV3.

### 3.2 SAV3-infection upregulates LIA gene expression *in vitro* while IPNV infection has limited effect

**Figure 3:**
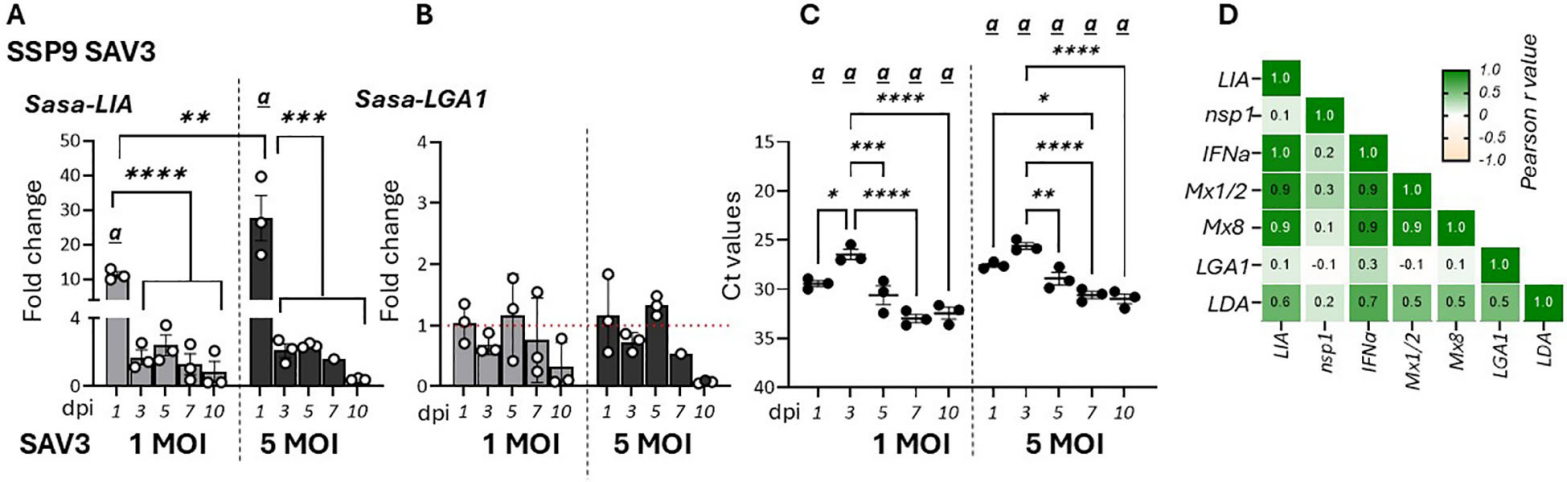
*Sasa-LIA* gene expression is transiently upregulated in SAV3 infected SSP9 cells. Relative gene expression of **(A)** *Sasa-LIA* and **(B)** *Sasa-LGA1* measure 1-10 days post SAV3 *in vitro* infection in SSP9 cells (MOI = 1 or MOI = 5). Bars represent mean ± SE (n = 3/time point with individual results shown as white dots) expressed as fold induction compared to non-infected cells at each time point. All samples were analyzed with RT-qPCR and normalized against EF1α as reference gene. **(C)** Individual Ct values and mean values of nsp1 at the different time points following infection. Significant (*p* < 0.05) upregulation compared to the corresponding control group is indicated by *(a)* and significant differences among the different time points are indicated, *p < 0.05, **p < 0.01, ***p = 0.0001, ****p < 0.0001. **(D)** Correlation matrix showing mRNA expression correlation of select genes. Numbers in boxes indicate R values of analyzed gene pairs and the strength of correlation is colored as depicted in the scale bar. (Relative gene expression of ache genes in the correlation matrix can be found in **Supplementary Figure 2**).

To further assess the effects of viral infection on the regulation of L lineage gene expression, specifically LIA and LGA1, we analyzed the temporal dynamics of these genes in response to SAV3 infection in SSP-9 cells (37). Similar to *in vivo* observations *Sasa-LIA* gene expression was upregulated during the early stage of infection (1 dpi) with infection using a higher virus MOI resulting in higher *Sasa-LIA* induction (**Figure 3A**). Further, despite highest detection of SAV nsp1 RNA at 3 dpi*, Sasa-LIA* transcriptional levels returned to near baseline by this time point and remained at this level throughout the course of the infection (**Figures 3A, C**). A similar gene expression pattern was observed for IFNa which was strongly correlated with that of *Sasa-LIA* (**Figure 3D**, **Supplementary Figure 2**). In contrast to what was observed following *in vivo* SAV3 challenge *Sasa-LGA1* was not significantly induced in response to SAV3 infection in SSP-9 cells (**Figure 3B**).

**Figure 4:**
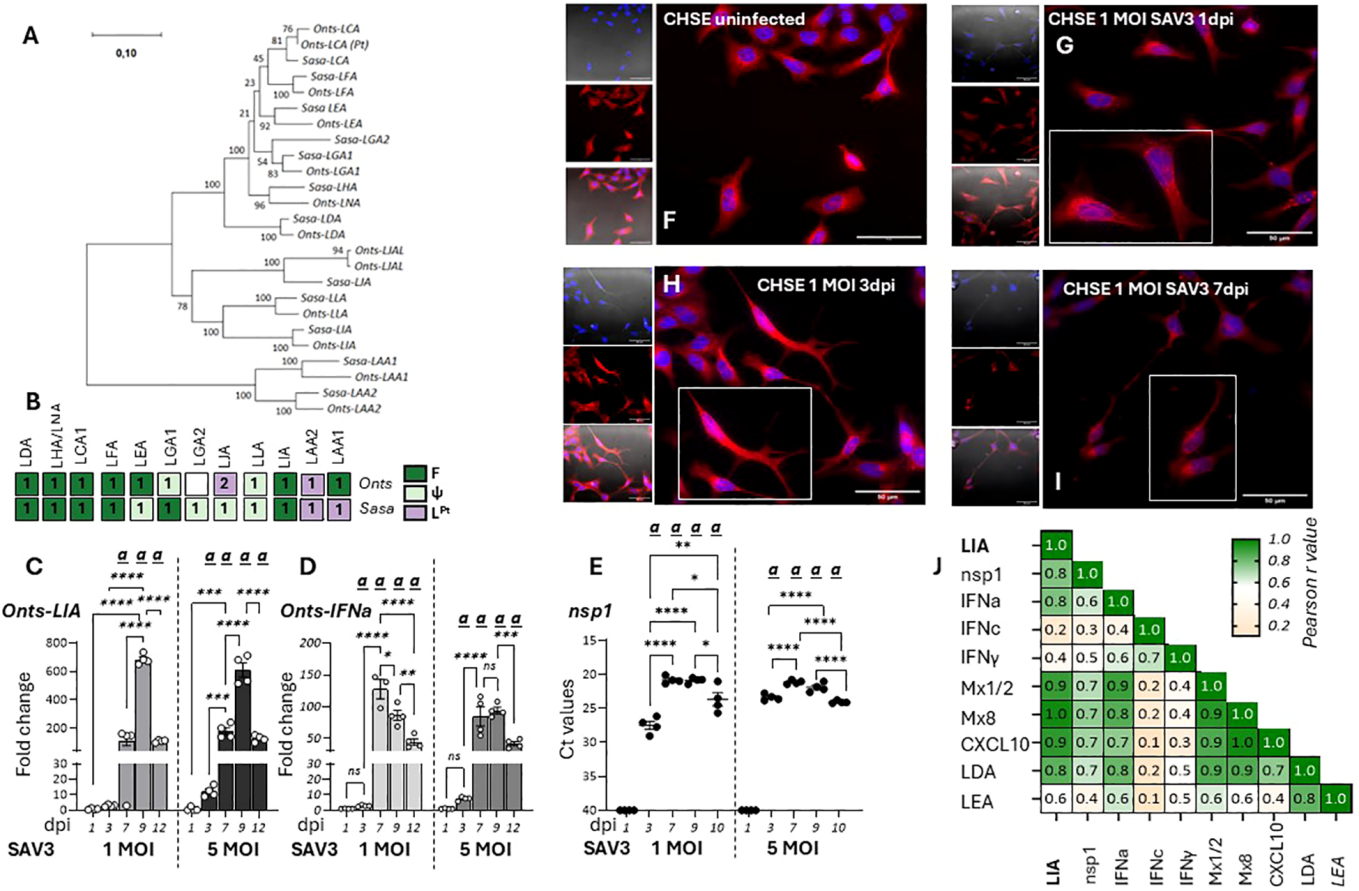
*Onts-LIA* is highly expressed in SAV3 infected CHSE cells. **(A)** Phylogenetic tree illustrating the relationships among Atlantic salmon and Chinook salmon L lineage genes. The tree with the highest likelihood is shown, and is drawn to scale, with branch lengths representing the number of substitutions per site. **(B)** Schematic representation summarizing the distribution and number of full length bona fide functional (**F**, dark green), pseudo (ψ, light green) and partial (L^Pt,^purple) L linegae genes in the two species. Relative gene expression of **(C)** *Onts-LIA* and **(D)** Onts-IFNa measure 1-12 days post SAV3 *in vitro* infection in CHSE cells (MOI = 1 or MOI = 5). Bars represent mean ± SE (n = 4/time point with individual results shown as white dots) expressed as fold induction compared to non-infected cells at each time point. All samples were analyzed with RT-qPCR and normalized against EF1α as reference gene. SAV3 progression was estimated by **(E)** individual Ct values and mean values of nsp1 at the different time points following infection and **(F-J)** deconvolution microscopy. Representative images of control **(F)** and 1 MOI SAV3 infected CHSE-WT at **(G)** 1 dpi **(H)**. 3dpi and **(I)** 7 dpi. Cells were stained with membrane stain CellMask™ Orange (red) and cytosolic stain NucSpot^®^ Live 488 (Nunc488) (blue). The scale bar indicates 50μm. Morphological distinctions (CPE) were detected in CHSE cells at 1, 3, and 5dpi. **(J)** Correlation matrix showing correlation of mRNA expression of select genes throughout the experiment. Numbers in boxes indicate R values of analyzed gene pairs and the strength of correlation is colored as depicted in the scale bar. Significant (*p* < 0.05) upregulation compared to the corresponding control group is indicated by *(a)* and significant differences among the different time points in **(C-E)** are indicated, *p < 0.05, **p < 0.01, ***p = 0.0001, ****p < 0.0001. The infection experiment was performed twice with reproducible results.

Next, transcriptional responses of L lineage gene expression in response to viral infections in the chinook salmon embryonic cell line CHSE-214 were investigated. Here it should be noted that L lineage gene profiles in salmonids is complex, with a remarkably high degree of species-specific adaptations both in gene numbers and functionality (12). Thus, the L lineage profile of chinook salmon was contrasted with that of Atlantic salmon, revealing that while LIA is highly conserved and present as a bona-fide functionally expressed gene in both species, LGA1 has become pseudogenised in Chinook salmon (**Figure 4A**, **Supplementary Figure 3**). Consistent with the genomic data no expression of *Onts-LGA1* were observed in CHSE-214 cells (data not shown). Accordingly, only *Onts-LIA* was examined further. Compared to SSP-9 cells, for CHSE-214 exposed to SAV3 at MOIs of 1 and 5 viral kinetics was different likely reflecting the genetic differences inherent to these two cell types. In CHSE-214 cells, *Onts-LIA* transcript levels were not significantly induced until around 7 dpi reaching a peak of induction at 9 dpi before declining by 12 dpi (**Figure 4C**). However, similar to what was observed in SSP-9 cells *Onts-LIA* mRNA levels were highly correlated with nsP1 and IFNa transcript levels, as well as with all representative interferon stimulated genes (ISGs) tested (Mx1/2, Mx8 and CXCL10) while no significant correlation was manifested between *Onts-LIA* and IFNc transcripts (**Figure 4J**, **Supplementary Figure 4**). When contrasted with the presence of SAV3 virus, as inferred from detection of nsp1 transcript levels, measured by RT-qPCR, viral transcripts could be detected already at day 3, reaching a peak at 7-9 dpi before declining at the 12 dpi time point (**Figure 4E**). Similarly, a gradual increase in CPE monitored via confocal microscopy on fixed cells, using cell mask and nuclear staining was detected for cells infected with 1 MOI SAV3 with signs of infection apparent as early as 1 dpi. Throughout the infection, compared to control cells, infected cells displayed distinct morphological changes including compromised cytoskeleton, cell membrane permeabilization, rounding and loss of cell adhesion (**Figures 4F-I**). In conclusion, these data indicate that while SAV3 infection leads to upregulation of *Onts-LIA* gene expression, transcriptional regulation appears to be tightly linked, possibly directly mediated by interferon stimulation rather than direct viral recognition.

**Figure 5:**
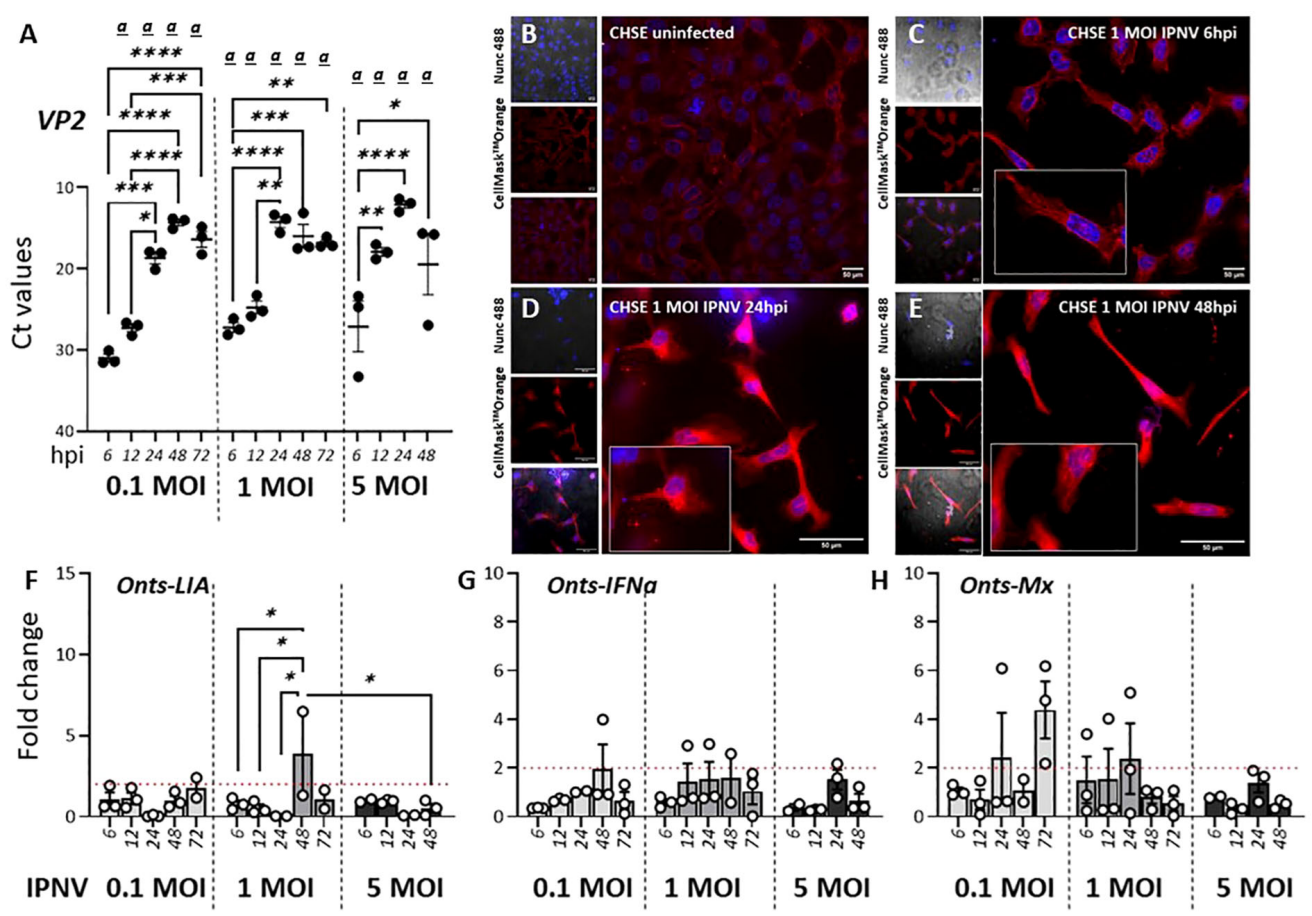
IPNV infection has a negligible effect on Onts-LIA expression. **(A–D)** Representative images of uninfected **(A)** and 1 MOI infected CHSE-214 cells at **(B)** 6 hours post infection (hpi) **(C)** 24 hpi and **(D)** 48hpi. Cells were stained with membrane stain CellMask™ Orange (red) and cytosolic stain NucSpot^®^ Live 488 (Nunc488) (blue). The scale bar indicates 50μm. Morphological distinctions (CPE) were detected in CHSE cells at 6, 24 and 48hpi, upscaled in inserts. **(E)** Detection of viral VP2 mRNA by RT-qPCR following infection with 0,1, 1 or 5 MOI IPNV. RNA extracted from cells at MOI 5 at 72 h was, because of extensive CPE, of poor quality and no data from this time point is therefore represented in the figure. The relative gene expression of **(F)** *Onts-LIA*, **(G)** Onts-IFNa1 and **(H)** Onts-Mx were measured 6–72 h post *in vitro* infection in CHSE-214 cells with IPNV (MOI = 0.1, 1 and 5). Bars represent mean ± SE (n = 3/time point with individual results shown as white dots) expressed as fold induction compared to non-infected cells. Significant (*p* < 0.05) upregulation compared to the corresponding control group is indicated by *(a)* and significant differences among the different time points in are indicated, *p < 0.05, **p < 0.01, ***p = 0.0001, ****p < 0.0001.

In contrast to SAV3 which has been shown to induce the expression of type I IFN and ISGs both *in vitro* and *in vivo* IPNV, a dsRNA virus, inhibits IFN-induced responses in CHSE-214 cells (46). An interesting question was therefore how infections with IPNV would potentially influence the expression of L lineage genes. For CHSE-214 cells exposed to 1 MOI IPNV, CPE monitored via confocal microscopy showed a progressive loss of cell adhesion and compromised cell membrane and cytoskeleton along with increasing levels of lysed cell debris (**Figures 5B–E**). Signs of cell stress was apparent already at 6 hpi and progressively increased throughout the course of the infection. In concordance with the microscopic analysis **Figure 5A** shows IPNV VP2 transcript levels in the infected cells, which increased over time, indicating a productive infection. However, in stark contrast to SAV3 infections of the same cells, no significant induction of *Onts-LIA* was observed as compared to control cells. The mRNA expression of IFNa and Mx paralleled *Onts-LIA* expression and in general, expression of all three genes were very low throughout the study.

### 3.3 Sasa/Onts-LIA and Sasa-LGA1 expression is differentially induced by representative type I and type II IFNs

**Figure 6:**
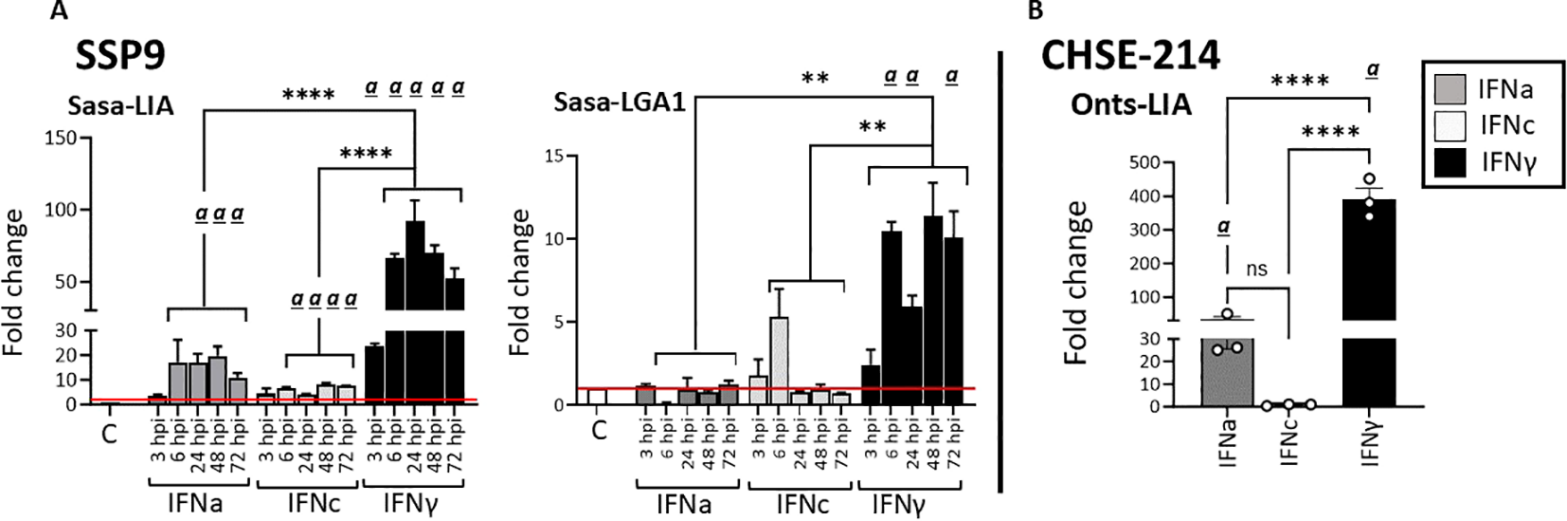
Type I (IFNa, IFNb and IFNc) and type II interferon stimulation results in variable induction of *Sasa-LIA* and *Sasa-LGA* in HKLs. Expression of MHC class I L lineage **(A)** *Sasa-LIA* and **(B)** *Sasa-LGA1* and in HKL stimulated with 500 U rIFNa (dark grey), 500 U rIFNc (light grey) 500 U rIFNb (white) or 10ng/ml rIFNγ (black) for 24. Gene expression data were normalized against the reference gene EF1α, and log 2-fold changes were calculated using the unstimulated sample (media alone) at the same time point. The data represent values from [n ϵ (4;8)] individuals, with each dot representing cells isolated from an individual fish. The line intersecting the y-axis at 1 represents the unstimulated control that the fold change of the treatments is in relation to. Significant (*p* < 0.05) upregulation compared to control (media alone) is indicated by *(a)* and asterisks indicates the strength of significance: **p < 0.01, ***p < 0.001, and ****p < 0.0001 among the indicted bars. The data presented is representative of two separate experiments.

**Figure 7:**
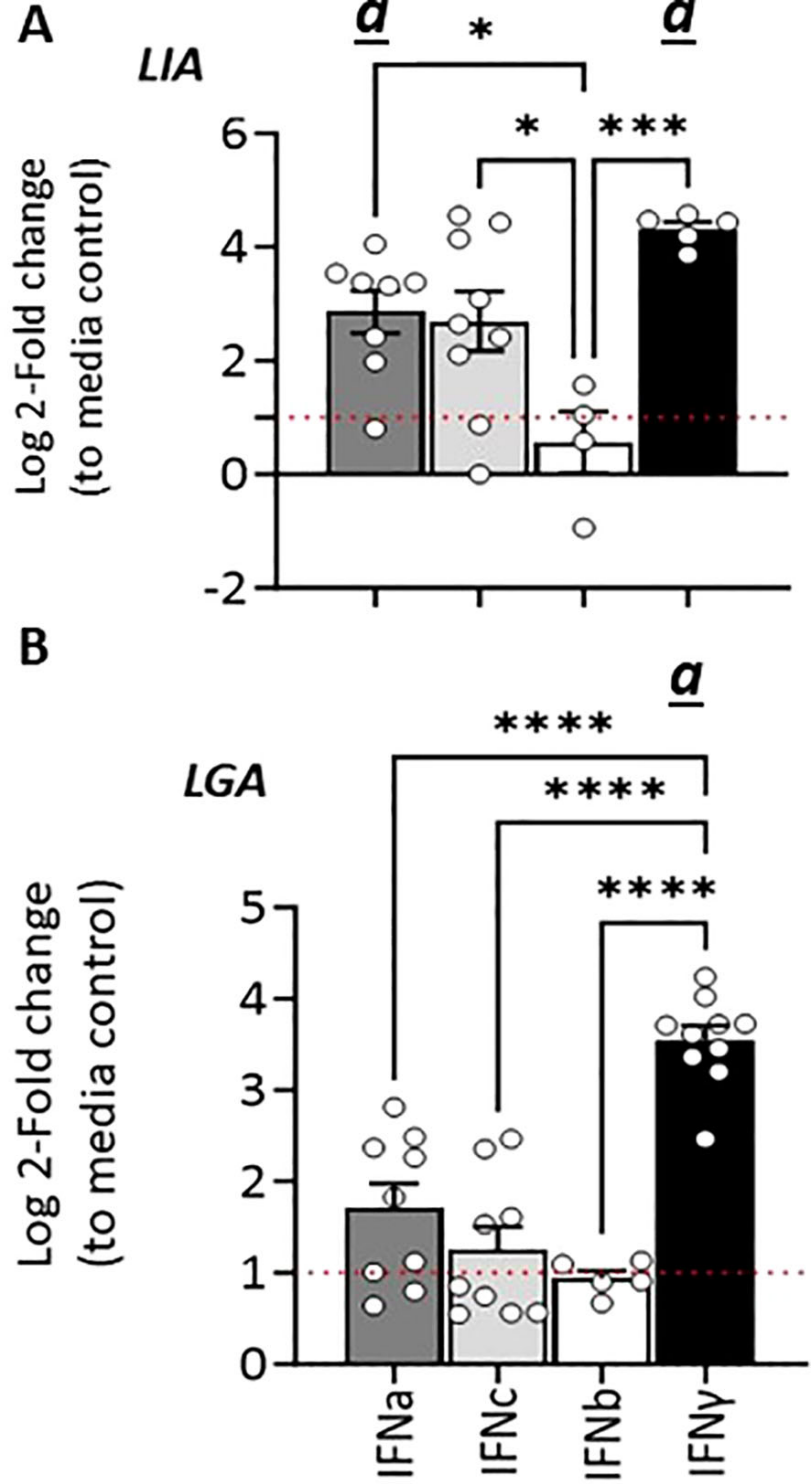
Stimulation with type I and type II IFNs differentially regulates *Sasa-/Onts-LIA* and *Sasa-LGA1* transcription in **(A)** SSP9 and **(B)** CHSE-214 cells. Expression of MHC class I L lineage *Sasa-LIA/Onts-LIA* and *Sasa-LGA1* in SSP9 and CHSE-214 cells 24 h post stimulation with 500U rIFNa (dark grey), 500U rIFNc (light grey) and 10ng/ml rIFNγ (black). Gene expression data at each time point were normalized against the reference gene EF1α and fold changes were calculated using control cells stimulated with media alone collected at the same time point. The line intersecting the y-axis at 1 represents the control that the fold change of the treatments is in relation to. Significant (*p* < 0.05) upregulation compared to the corresponding control group is indicated by *(a)* and Asterisks indicates the strength of significance between the indicated time points and are indicated by *p < 0.05, ***p = 0.0001, ****p < 0.0001. The data presented is representative of four separate experiments.

Given that type I IFN classes are known to differ in their responses, we compared the ability of recombinant representatives of group 1 (rIFNa1) and group II (rIFNb and rIFNc) type I IFNs to modulate L lineage gene expression in primary head kidney leucocytes (HKLs, **Figure 6**), SSP-9 (**Figures 7A, B**) and CHSE-214 cells (**Figure 7C**). Further, to obtain additional insight into the interplay among type I and type II interferon induction and transcription regulation of L lineage genes, the modulating abilities of representative type I IFNs was contrasted with that of recombinant type II IFN (rIFNγ). For HKLs, expression of the interferon stimulated genes Mx1/2 and Mx8, which have been shown to differentially respond to IFNa and IFNγ stimulation (30) were included as controls and the transcriptional induction of IL-1, TNFα, IFNa1 and IFNγ was investigated in parallel (**Supplementary Figure**). In HKLs, 12 hours post stimulation, both *Sasa-LIA* and *Sasa-LGA1* were strongly (~16 fold) upregulated in response to rINFγ stimulation (**Figures 6A, B**). *Sasa-LIA* expression was also significantly upregulated in response to type I rIFNa1 and rIFNc stimulation, while Sasa-LGA1 showed a modest induction in response to rIFNa1 and no significant induction in response to rIFNc stimulation at this time point. Comparably, while rIFNb stimulation induced expression of Mx1/2 and Mx8 at comparable levels to that of rIFNa1, no induction of L lineage gene expression was observed in response to rIFNb stimulation (**Figure 6**, **Supplementary Figure 5**). A modest induction of *Sasa-LHA* gene expression was observed in response to rINFγ with no significant induction in response to any of the type I IFNs tested. *Sasa-LDA* did not respond to type I IFNs or type II IFN stimulation and neither *Sasa-LCA* nor *Sasa-LFA* transcripts were detected in HKLs above threshold levels. Comparing the induction kinetics of *Sasa-LIA* and *Sasa-LGA1* genes revealed that both L lineage genes responded rapidly, albeit with different magnitudes and distinct kinetics following stimulation with either rIFNa1, rIFNc or rIFNγ (**Supplementary Figure 5**). Significantly elevated transcript levels were detected as early as 6 hps, peaking between 12 and 24 hps, followed by a return to baseline by 48hps. Induction kinetics were different depending on the stimuli, with rIFNa1 resulting in a somewhat bimodular upregulation peaking at 6 and 24hps. Comparably, highest transcription levels in response to INFc was detected at 6 hps for both *Sasa-LIA* and *Sasa-LGA1* while IFNγ stimulation resulted in continuous increasing levels of *Sasa-LIA* up on till the 24h time point while *Sasa-LGA1* transcription levels peaked at the 6-hour time point.

Similarly, in SSP-9 cells stimulated with rIFNγ we observed a rapid and marked upregulation of *Sasa-LIA* expression, a > 20-fold increase in gene expression was observed within 3 hps that peaked (>90-fold) at 24 h post-treatment and then declined somewhat at the last time point analyzed (72 h post-treatment, **Figure 7A**). Similarly, rIFNa1, and to a lesser extent rIFNc stimulation upregulated *Sasa-LIA* expression, albeit at a significantly lower level compared to rIFNγ. These results were largely recapitulated in CHSE-214 cells where we observed marked induction of *Onts-LIA* in response to stimulation with both rIFNγ and rIFNa1 but not to rIFNc (**Figure 7C**).

In SSP-9 cells the highest level of *Sasa-LGA1* induced expression, with a >10-fold induction compared to unstimulated cells, was observed in response to rIFNγ stimulation while rIFNc stimulation resulted in peak of *Sasa-LGA1* expression at 6 hps that declined by the 24 h time-point (**Figure 7B**).

### 3.4 Pharmacological inhibition of the JAK/STAT pathway reduces rIFNa1, rIFNc and rIFNγ-induced Sasa-LIA and Sasa-LGA1 lineage expression

**Figure 8:**
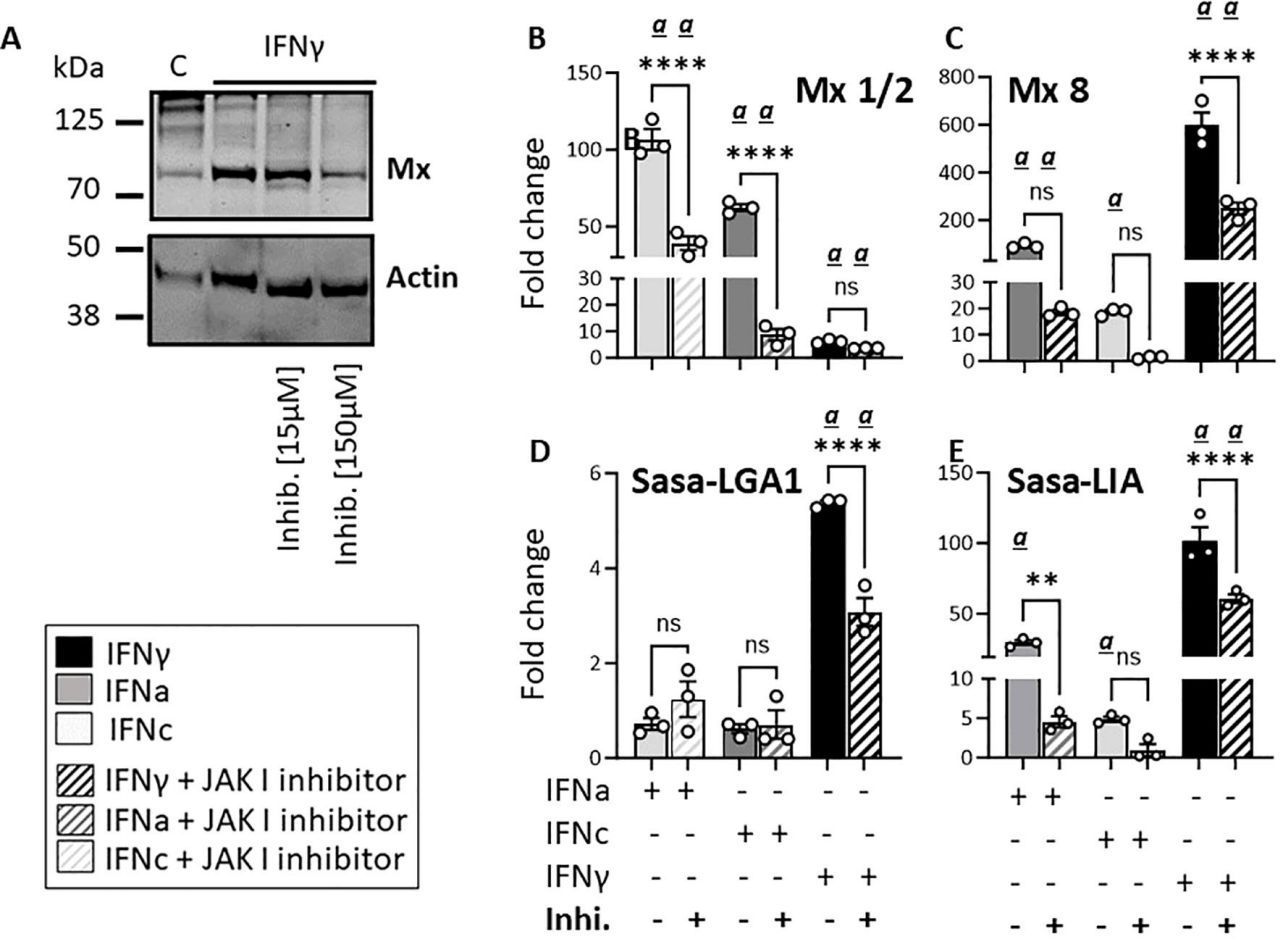
Pharmacological inhibition of the JAK/STAT pathway reduces rIFNa1, rIFNc and rIFNγ-induced *Sasa-LIA* and *Sasa-LGA1* lineage expression in SSP-9 cells. **(A)** Western blot of SSP-9 cell lysats harvested from cells stimulated for 24h in the presence or absence of 15nM or 150 nM JAK 1 inhibitor. Unstimulated SSP9 cells are included as a control. Gene expression of **(B)** Mx1/2, **(C)** Mx8, **(D)** *Sasa-LGA1* and **(E)** *Sasa-LIA*, in SSP-9 cells stimulated for 16 hours with 500U IFNa, 500U IFNc or 10ng IFNγ, in the presence (striped bars) or absence (solid bars) of 15nM JAK 1 inhibitor (In SolutionTM JAK Inhibitor; Calbiochem). Gene expression data at each time point were normalized against the reference gene EF1αB and fold changes were calculated using media with inhibitor as reference. Each dot represents an individual fish. The data represent values from [n ϵ (3)] technical replicates and the data presented is representative of three separate experiments. Asterisks indicate significant reduction in upregulation with inhibitor compared to respective controls. Significant (*p* < 0.05) upregulation compared to the corresponding control group is indicated by (α) and significant differences among the different timepoints are indicated, ** *p*< 0.01, ****p < 0.0001, ns, non-significant differences.

To further assess the impact of type I and type II IFN stimulation on L lineage gene induction, we analyzed Sasa-*LIA* and *Sasa-LGA1* expression in SSP-9 cells in the presence or absence of a JAK inhibitor I (a reversible ATP-competitive inhibitor of Janus protein tyrosine kinases (JAKs)). As expected, inhibiting the JAK/STAT pathway resulted in a reduction in IFN type I induced Mx1/2 and IFNγ induced Mx8 gene expression and at a 10-fold higher dose the IFNγ-induced Mx protein levels were visibly affected (**Figures 8A-C**). **Figures 8D, E** shows that *Sasa-LGA1* and *Sasa-LIA* transcript levels significantly decreased in rIFNγ -treated cells in the presence of the inhibitor compared to control cells. Reduction in *Sasa-LIA* transcript levels in the presence of the inhibitor were also apparent in rIFNa1-treated and, albeit not significant, in SSP-9 cells stimulated with rIFNc. Notably, no significant effect of the inhibitor was observed with regard to the basal expression of either *Sasa-LIA* or *Sasa-LGA1* (**Supplementary Figure 6**).

Similarly, following a 12-hour incubation with 15nM JAK I inhibitor a marked reduction in rIFNγ as well as rIFNa and rIFNc induced upregulation of both *Sasa-LIA* and *Sasa-LGA1* was observed in HKLs (**Supplementary Figure 7**). Collectively, these data indicate that *Sasa-LIA* and *Sasa-LGA1* interferon induced transcription occurs, at least partially, in response to type I IFN and type II IFNγ induced JAK/STAT dependent signaling.

### 3.5 Identification of distinct regulatory promoter elements governing Sasa-LIA and Sasa-LGA1 transcription

**Figure 9:**
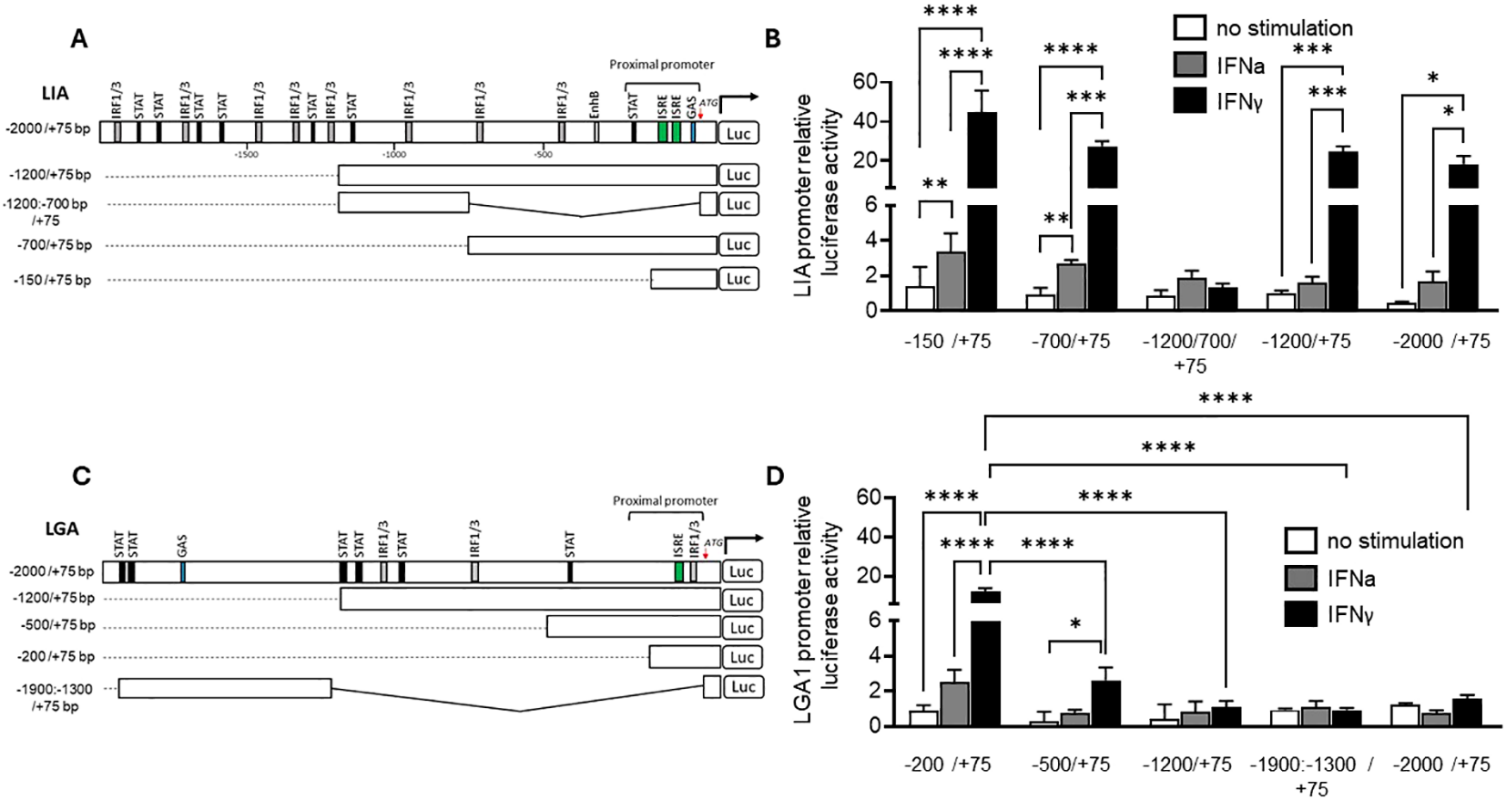
Transcriptional regulation of LIA and LGA1. Schematic presentation of WT and truncated **(A)** *Sasa****-****LIA* and **(C)** *Sasa-LGA* promoter constructs connected with luciferase reporter. Select positions in the 5`-flanking regulatory sequence of LIA and LGA, ISRE (GAAA-N2-GAAA), Gas-like (TTCAGAA), STAT and IRF1/3 core containing (GGAA or TTCC) regulatory sequences are shown, the ATG start codon is designated by +1. **(B)** Luciferase activity of WT and four truncated LIA luciferase promoters; LIA:-2000/+75 bp, LIA:-1200/+75 bp, LIA:-1200/-700/+75 bp, LIA:-700/+75 bp and LIA:-150/+75 bp following stimulation with 600U rIFNa-1 (dark grey), 30ng IFN-γ (black) or unstimulated (white). **(D)** Luciferase activity of WT and four truncated LGA luciferase promoters LGA:-2000/+75 bp, LGA:-1200/+75 bp, LGA:-500/+75 bp, LGA:-200/+75 bp and LGA:-1900/-1300/+75 bp following stimulation with 600U rIFNa-1 (dark grey), 30ng IFN-γ (black) or unstimulated (white). Data were reported as mean ± SE from three independent experiments performed in quadruplictes. *p < 0.05, *** p<0.001, **** p<0.0001.

**Figure 10:**
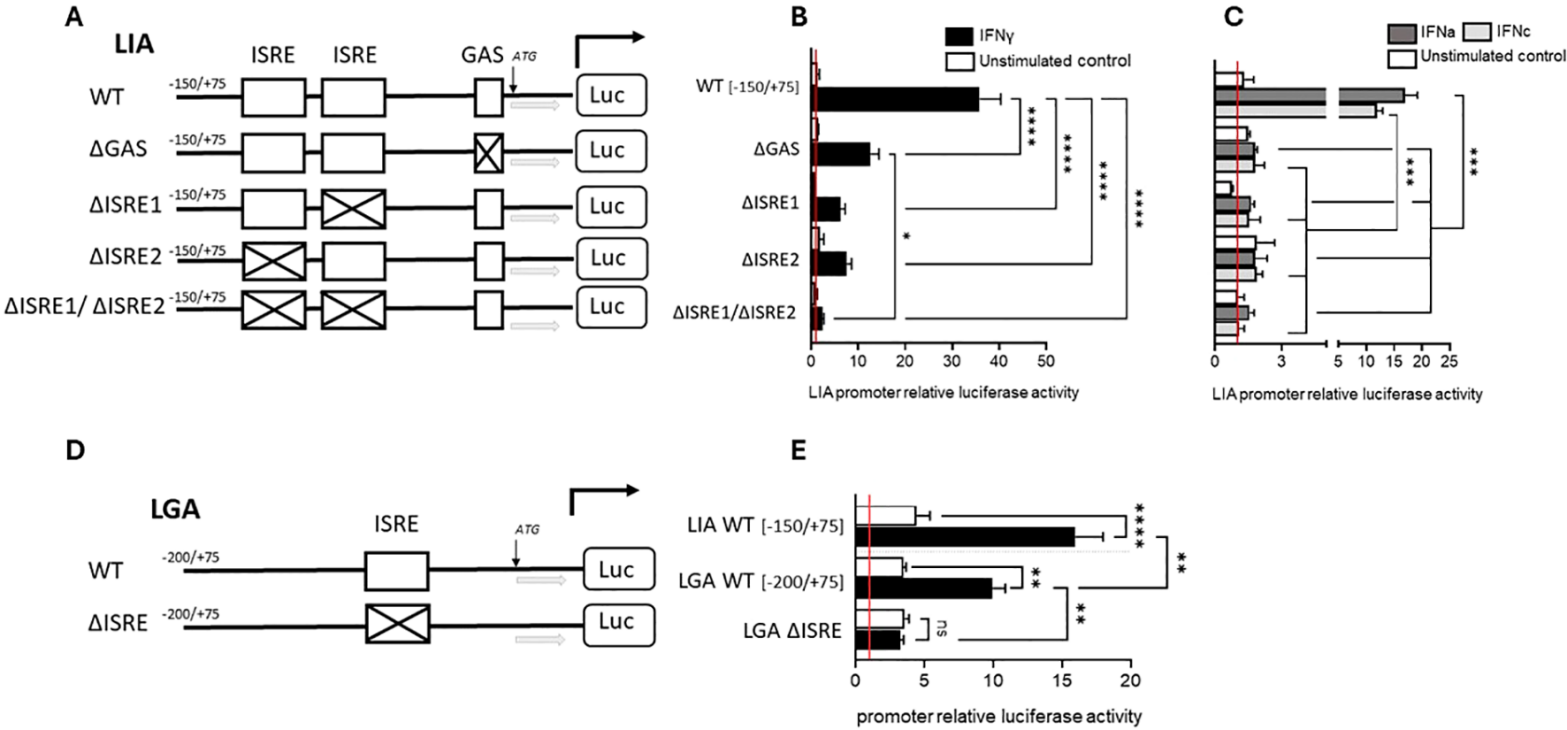
Induced expression of LIA and LGA1 is differentially dependent on canonical and noncanonical interferon response elements. **(A)** Schematic presentation of four LIA promoter mutants, ΔGAS, ΔISRE1, ΔISRE2, and ΔISRE1ΔISRE2 (LIA pro–ΔGAS-luc, LIA pro–ΔISRE1-luc, LIA pro–ΔISRE2-luc, LIA pro–ΔISRE1ΔISRE2-luc), all mutants are based on the -150/+75bp WT proximal LIA promoter region. **(B)** Luciferase activity of the four LIA mutants, ΔGAS, ΔISRE1, ΔISRE2, and ΔISRE1ΔISRE2, following stimulation 30 ng IFN-γ (black) or unstimualted (white) and **(C)** following stimulation with 600U Type I IFNa-1 (dark grey) and 600U rIFNc (light grey) or unstimulated (white). **(D)** Schematic presentation of WT LGA:-200/+75 and LGA pro-ΔISRE-luc mutant. **(E)** Luciferase activity of LIA:-150/+75 bp, LGA:-200/+75 bp promoter, pro–ΔGAS-luc following stimulation with 30ng IFN-γ (black) or unstimulated (white). Data were reported as mean ± SE from three independent experiments performed in quadruplicates. *p < 0.05, **p < 0.01, *** p<0.001, **** p<0.0001.

The promoter regions of Sasa-LIA and Sasa-LGA1 possess distinct binding sites (**Figure 9**). Within the proximal promoter region, spanning 500 bp upstream of the start codon canonical interferon response elements can be found in both *Sasa-LIA* and *Sasa-LGA1*. For *Sasa-LIA*, this region includes a putative STAT, two identical ISRE (GAAA-gt-GAAA), and a GAS-like (TTCAGAA) element while in the same region a single ISRE (GAAA-ga-GAAA) and a IRF1/3 site can be identified in *Sasa-LGA1*. Further, the distal promoter region (-2000/-500) of *Sasa-LIA* contains multiple putative STAT and IRF1/3 binding sites, the majority of which are concentrated in the -2000 to -1000bp region while the distal promoter region of *Sasa-LGA1* contains a GAS-like element (TTCAGAA) and a concentration of STAT and IRF1/3 elements (**Figure 9**, **Supplementary Figure 8**). The promoter activity of *Sasa-LIA* and *Sasa-LGA1* was analyzed through luciferase assay in CHSE-214 cells (**Figure 9**). For *Sasa-LIA*, by stepwise truncating the promoter sequences it was apparent that the construct containing the two tandemly located ISRE and the GAS-like element (-150/75-Sasa-LIA) retained maximum luciferase activity, substantiating the assumption that ISRE and or GAS elements are essential for IFNγ, IFNa and IFNc induced expression of LIA in salmonids (**Figures 9A, B**). Thus, the (-150/75-Sasa-LIA) promoter construct was used in subsequence assays. Four mutated variants of Sasa-LIA -150/75 were synthesized, each containing mutations in either GAS (LIA-ΔGAS), ISRE1(LIA-ΔISRE1), ISRE2 (LIA-ΔISRE2), or double mutations for ISRE1/2 regions (LIA-ΔISRE1/ΔISRE2) (**Figure 10A**). Upon transient transfection into CHSE-214 cells and subsequent stimulation with rIFNa1, rIFNc or rIFNγ, all four mutated constructs exhibited significantly reduced luciferase activity compared to the unmutated -150/75-Sasa-LIA construct (**Figure 10B**). Following rIFNa1 and rIFNc stimulation luciferase activity was completely ablated in all four mutants when compared to -150/75-Sasa-LIA with no significant difference in activity observed across the different mutated constructs (**Figure 10C**). Comparably, following rIFNγ stimulation the luciferase activity of LIA-ΔGAS, LIA-ΔISRE1 and LIA-ΔISRE2 constructs remained elevated compared to controls but dropped significantly, reducing the activity on average to ~44%, 22%, and 20% respectively compared to the wild-type construct. In comparison the LIA-ΔISRE1/ΔISRE2 construct showed no detectable activity above controls. These findings demonstrate that while optimal IFNγ induced expression of *Sasa-LIA* likely involves all three promoter motifs it is critically dependent on the two ISRE motifs. In comparison in *Sasa-LGA1* which has an arguably simpler active regulatory region, consisting of a single ISRE motif located 72 bp upstream of the start codon, no activity was observed for the LGA–ΔISRE1 construct indicating that the identified ISRE motif alone is essential for IFNγ–induced expression of Sasa-LGA1 (**Figures 10D, E**). Notably, while IFNγ stimulation significantly upregulated luciferase activity for the truncated Sasa-LGA1 -200/75 construct, *Sasa-LGA1* constructs consisting of the extended distal promoter regions, despite containing an intact ISRE motif, showed significantly reduced or no luciferase activity (**Figures 9C, D**).

### 3.6 Evolutionary conservation of interferon response promoter motifs across salmonid LIA and LGA1 sequences

**Figure 11:**
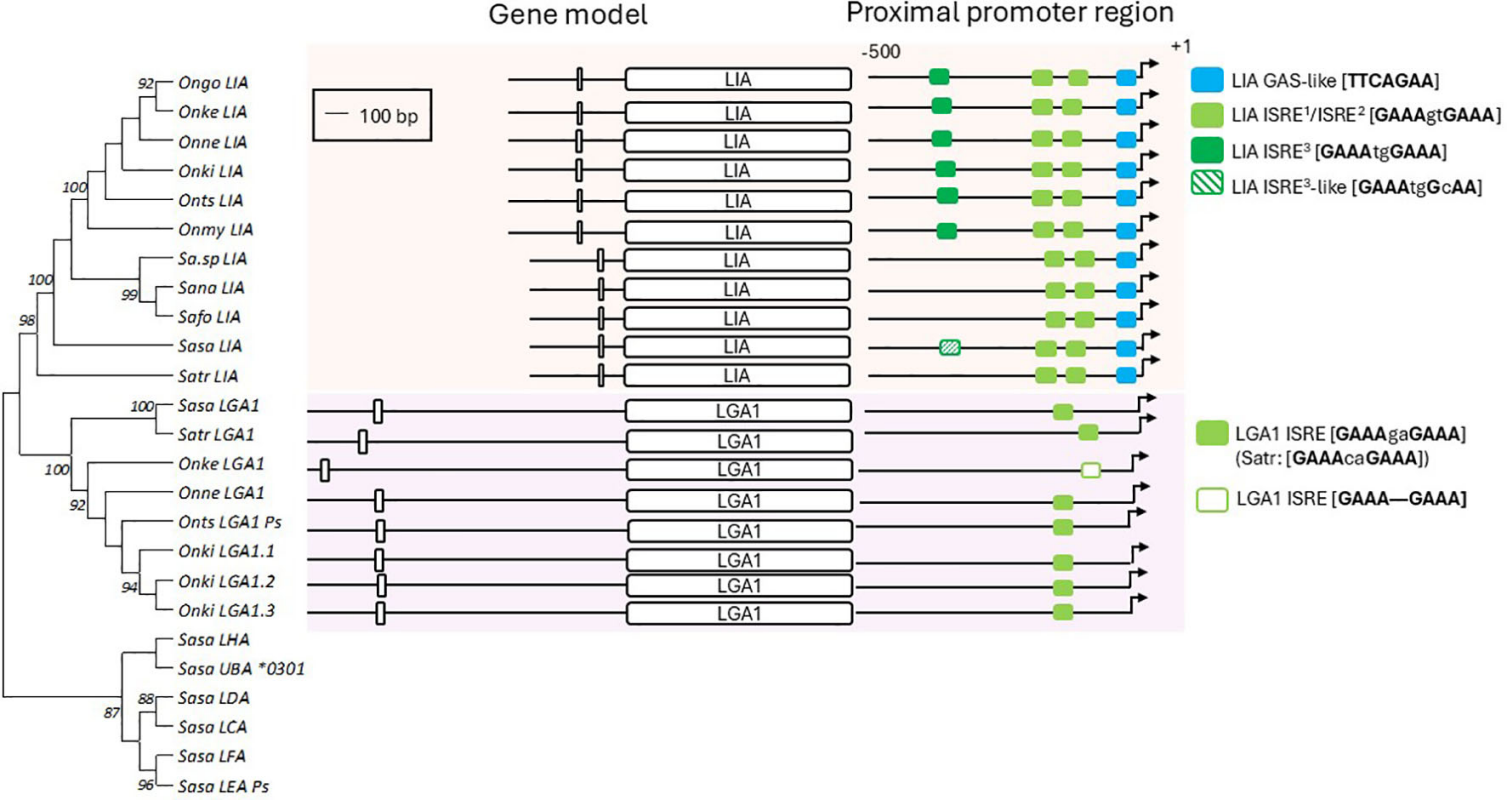
Promoter motifs and gene structure of LIA and LGA1 genes in representative salmonid species. **(A)** Evolutionary tree of L lineage promoter sequences from selected salmonid species. The tree was inferred using the Maximum Likelihood method and JTT matrix-based model with a bootstrap of 500 replicates. The tree with highest likelihood is shown, and is drawn to scale, with branch lengths representing the number of substitutions per site. The percentage of trees in which the associated taxa cluster together is indicated when >80. **(B)** schematic representation of intro-exon organization of LIA and LGA1 genes. Boxes indicate exons, separated by introns. **(C)** Schematic representation of the proximal promoter regions for LIA and LGA1 genes. Regulatory elements are indicated as colored boxes and arrows indicate transcriptional initiation sites.

LIA represents an evolutionary old L lineage gene (12) and LIA orthologs can be found as single gene copies in all 11 salmonid species with annotated genome assemblies currently available in the NCBI databases (data not shown). In comparison LGA1 sequences, based on phylogenetic clustering with previously identified L lineage gene sequences, were identified in six of the eleven salmonid species analyzed and display variation both in gene copy number and degree of pseudogenization. Phylogeny of the LIA and LGA1 proximal promoter region sequence, with strong bootstrap values, cluster based on subgroups forming two clades that are further supported by identification of key promoter elements and conservation of gene structure (**Figure 11**). The putative promoters of LIA all included a GAS-like element, with the consensus sequence TTCAGAA within 2-9 bp upstream of the ATG start codon and a minimum of two ISRE elements. In addition, a third putative ISRE sequence differing from the previously identified elements in the spacing nucleotides (GAAA-*tg*-GAAA compared to GAAA-*gt*-GAAA)) was identified in all six members of the *Onchorhynchus* genes but not in the *Salmo* nor *Salvelinus* LIA gene sequences (**Figure 11C**). Whether or not this is a functional ISRE element needs to be determined by functional assays. Other potential regulatory elements, including binding sites for the CCAAT/Enhancer Binding Protein β (C/EBPβ) in *Sasa-LIA* were not conserved across promoter regions identified in other species. Similarly, in all LGA1 promoter regions, with the exception of *Onke*-LGA1, a single consensus ISRE element was present. Collectively these data suggest that type I and type II IFN regulation of LIA and LGA1 gene expression is conserved across salmonids underpinning the roles of these non-classical MHC class I genes in anti-viral immunity.

## 4 Discussion

Among the numerous teleost non classical MHC class I genes, those belonging to the L lineage display a remarkable degree of species specific diversification with large variations in gene number and functionality, even among closely related species (12). This, coupled with unique, subgroup specific, constitutive and inducible expression patterns (15) indicates a large functional diversity, distinct from that of classical MHC class I. Collectively these observations imply that different L lineage genes possess specialized roles for combating specific types of pathogens. LIA and LGA1 in particular have emerged as potential key players in anti-viral defenses in salmonids (15). In this study, we present a systematic assessment of the complex transcriptional regulation underlying the inducible expression patterns of these genes revealing that while *LIA* and *LGA1* are both induced following SAV3 infection and interferon stimulations, each gene is uniquely regulated by evolutionary conserved, subgroup specific interferon response elements.

Salmonid *LIA* 5`-regulatory regions contain the highest number of interferon responsive elements among all L lineage genes examined to date, including a minimum of two canonical ISRE elements and a GAS-like sequence (5`-TTCaGAA-3`) within the proximal promoter region. This GAS-like motif which, depending on the species, is located -3 to -9 base pairs from the start codon, is different from previously investigated salmonid (rainbow trout) GAS-elements (TTC-n_3-4_-GAA or TTN-cnn-NAA (47),) which adhered more closely to the small palindromic consensus sequence typically defining a GAS element (48). Consistent with LIA mRNA expression patterns in primary HKLs, SSP-9 and CHSE-214 cells, Sasa-LIA reporter construct containing both ISRE and GAS-like elements showed strong reporter activity in response to rIFNγ stimulation and moderate to low activity following stimulation with two types of type I IFNs, rIFNa1 and rIFNc respectively. Targeted mutation studies show that while all three of the predicted interferon response elements are required for optimal reporter activity, type I IFN induction is critically dependent on a collaborative effort, as deleting any one of the three active promoter elements resulted in a complete ablation in luciferase activity. In contrast, while the GAS-like element is required for optimal IFNγ induced LIA transcription, significant promoter activity is retained even in the absence of a functional GAS element following stimulation. Comparably, mutating either of the two ISRE elements, which appear to contribute to transcriptional induction at roughly equal measures, resulted in a LIA promoter construct that failed to induce luciferase activity in response to rIFNγ. This is consistent with reporter construct studies in rainbow trout fibroblast cells examining the activity of three IFNγ induced genes containing both ISRE and GAS-elements demonstrating that, unlike what is typical for mammals, IFNγ induced promoter activity was dependent on the presence of ISRE- rather than canonical GAS elements (47). Thus, the full importance of GAS and/or GAS-like elements in the promoters of IFNγ responsive genes in fish remains unclear and induction of IFNγ induced genes may be strongly dependent on the induction of transcription factors, such as IRF1 which, alone or in combination with STAT1 has been suggested to play the role of a master regulator following IFNγ stimulation in Atlantic salmon (16).

Notably, while the proximal *Sasa-LGA1* promoter, similar to *Sasa-LIA* showed ISRE dependent luciferase activity in response to IFNγ stimulation, *Sasa-LGA1* promoter activity was, despite the presence of a canonical ISRE element, markedly reduced with the stepwise inclusion of more 5`located sequence stretches. Thus, indicating that the distal *Sasa-LGA1* promoter, in contrast to the corresponding region in Sasa-LIA, have *cis*-acting suppressive elements. This type of dual promoter is not unprecedented in the regulation of non-classical MHC class I genes. For example, expression of the human CD1d gene is regulated in a similar manner and is governed by a cell type specific promoter that contains both activating and repressing elements. Within the proximal promoter region, transcriptional regulators such as SP1, ETs families and all-trans-retinoic acid enhance promoter activity and drive CD1d expression (7). Conversely, lymphoid enhancer-binding factor 1 (LEF-1) binding elements situated within the distal promoter region have suppressive effect on CD1d promoter activity, negatively impacting CD1d transcript levels (8). LEF-1, together with other cis regulatory elements, functions as a key mediator of the Wnt signaling pathway, an evolutionarily conserved signaling pathway whose dysregulation has been associated with tumors, including hematological malignancies (49). Notably, putative LEF-1 binding elements (5′-CTTTGAA-3′) are present in all salmonid *LGA1* promoter sequences examined, (−777 to −771 in *Sasa-LGA1*) but are not found within the *LIA* promoter regions. Here, given the observed discrepancy with regard to presence of consensus ISRE and GAS elements in the promoters of L lineage genes (such as *LFA* and *LHA (* 15 *)*,) that, to date, do not show marked transcriptional induction in response to either SAV3 infection, type I or type II IFN stimulation it is not unlikely that suppressive and/or additional regulatory elements are involved in controlling transcription of these genes. Further, despite the strong interferon inducible activity of the *Sasa-LIA* promoter, *LIA* upregulation *in vivo*, unlike that of *LGA1*, is transient. Following SAV3 challenge *Sasa-LIA* expression, despite the continued presence of viral mRNA as well as elevated mRNA levels of IFNγ, type I IFNa and IFNc in the same sample returns to near baseline by 4 wpi indicating the activity of, as of yet, unidentified, transcriptional regulatory events. This transient expression pattern might imply that induction of LIA, which is normally expressed at low levels, have functional implications for the cell that, while favorable in the early stages of infection, are not beneficial if maintained elevated over time.

While it is clear that more studies are needed to elucidate the full regulatory programs of the various MHC class I L lineage genes it is apparent that there is a tight interplay among LIA and LGA1 induction and the interferon response. Both LIA (*Sasa-LIA* and *Onts-LIA*) and *Sasa-LGA1* overall respond strongly to rIFNγ, although the level and temporal dynamics of the response were, depending on the cell population investigated, different. Thus, similar to what has been reported for mammalian non-classical MHC class I genes and proteins ( (2, 3, 11)), a certain degree of cell type specific regulation with regard to L lineage genes may be inferred. Less potent compared to rIFNγ, different type I IFNs groups also enlist unique L lineage gene induction patterns. Both type I rIFNa1 and to a lesser extent type I rIFNc induce LIA transcription while type I IFNs only weakly upregulate *Sasa-LGA1* mRNA levels. Comparably, no induction of any of the six examined L lineage genes were observed in HKLs in response to rIFNb which might reflect a less potent antiviral activity previously documented for this specific interferon group (25, 26). The reliance on an interferon response for transcriptional induction of LIA is further supported by the lack of significant upregulation of *Sasa-LIA* and *Onts-LIA* respectively in IPNV infected CHSE-214 cells. It is tempting to speculate that this lack of induction reflect the weak type I IFN response induced by this particular virus in these cells (46). However, these observations are preliminary. Many viruses are known to suppress MHC expression both indirectly by targeting IFN induced signaling pathways (50) and directly as exemplified by HMCV induced proteolytic degradation off MICA in an attempt to avoid NK cell mediated cytotoxicity (51, 52) thus, it is possible that IPNV interfer with *Onts-LIA* expression in a more direct manner. Undoubtelly, more studies, including *in vivo* challenges will be needed to fully elucidate a potential role of Sasa-LIA, and by extension other L lineage genes, in anti-viral immune responses against IPNV. However, given the demonstrated roles of type I and type II IFNs in regulating the transcriptional induction of LIA and LGA1 it is likely that upregulation of these genes upon SAV3 infection is, in large parts due to the type I (IFNa and IFNc) and/or type II induction observed in the same samples. Consistent with this, while, as assessed by nsp1 mRNA levels in hearts, there are no significant differences in viral burden among wild-type and attenuated infectious rSAV3 strains there is significantly lower induction of *Sasa-LIA* in heart from the rSAV3-E2_319A_ infected fish. In parallel, IFNa expression at the same time point and in the same sample was visibly lower compared with the other groups injected with infectious virus, possibly accounting for the suboptimal induction of *Sasa-LIA*. In comparison, no significant differences in expression of IFNγ or *Sasa-LGA* were observed between the groups infected with either rSAV3, rSAV3-E2_319A_ or rSAV3-CapNLS supporting the predominant reliance on type II IFNγ for optimal *Sasa-LGA1* induction. Somewhat surprisingly, at 4 wpi no significant difference in LGA1 expression were found between the group injected with inactivated virus compared to those infected with infectious virus. Similarly, previous studies on the same material have shown that while there is no detectable nsp1 mRNA nor a detectable induction of Mx in fish injected with inactivated SAV3 virus, significant upregulation of another interferon stimulated gene, viperin was detected (34). Thus, while this study provides compelling evidence that interferon, type I and type II, driven induction of *Sasa-LIA* and *Sasa-LGA1* occurs as a downstream result of engagement of specific interferons with their appropriate interferon receptor and subsequent activation of the JAK-STAT signaling pathway there is also a potential for IFN signaling independent L lineage upregulation. Such flexibility in non-classical MHC class I L lineage receptor expression likely allows for fine-tuned immune responses in different microenvironments and in response to different immunological challenges.

In addition to different IFN induction potentials and distinct temporal transcriptional patterns LIA and LGA1 also display distinct evolutionary patterns. While *Sasa-LIA* has clear orthologs in all salmonid species analyzed to date and can be traced as far back as Northern pike (*Esox lucius*), which is a basal sister clade to salmonids, the presence of a bona fide *LGA1* gene is sporadic across various species (12). In some salmonid species, exemplified by Chinook salmon (*O*. *tshawytscha*), a single clearly defined *LGA1* gene is present but has been rendered unfunctional by the presence of a stop codon in the beginning of the C-like domain. While this opens questions with regard to the biological relevance of *LGA1* it also hints to the possibility that, within the large and highly species-specific L lineage gene family, there are instances of gene redundancy, functional cooperation and/or functional overlap. Chinook salmon, similar to Atlantic Salmon, have multiple functionally expressed L lineage genes, and it could be that, in lieu of a functional *LGA1* gene another gene performs a similar role as that attributed to *Sasa-LGA1* in Atlantic salmon. This is in stark contrast to *LIA*, where the gene is not only present in all species but, based on transcriptional regulatory responses in cells derived from Atlantic salmon as well as chinook salmon, appear to have retained a conserved function indicating an important role of LIA in antiviral responses across, and possibly even beyond salmonid species.

In conclusion, while it is not unlikely that pattern recognition receptor signaling may impact the transcription of *LIA*, *LGA1*, and potentially other L lineage genes, it is nevertheless clear that these genes are induced following exposure to distinct interferons, of which IFNγ is the most potent. This work is a step towards understanding what is emerging to be complex and interconnected immune functions of non-classical MHC class I L lineage genes in Atlantic salmon in particular and bony fish in general.

## Data Availability

The original contributions presented in the study are included in the article/**Supplementary Material**; further inquiries can be directed to the corresponding author/s.

## References

[1] Adams EJ Luoma AM. The adaptable major histocompatibility complex (MHC) fold: structure and function of nonclassical and MHC class I-like molecules. Annu Rev Immunol. (2013) 31:529–61. doi: 10.1146/annurev-immunol-032712-095912 23298204

[2] Dougan SK Kaser A Blumberg RS. CD1 expression on antigen-presenting cells. Curr Top Microbiol Immunol. (2007) 314:113–41. doi: 10.1007/978-3-540-69511-0_5 17593659

[3] Lamichhane R Ussher JE. Expression and trafficking of MR1. Immunology. (2017) 151:270–9. doi: 10.1111/imm.2017.151.issue-3 PMC5461101 28419492

[4] Ludigs K Seguín-Estévez Q Lemeille S Ferrero I Rota G Chelbi S. NLRC5 exclusively transactivates MHC class I and related genes through a distinctive SXY module. PloS Genet. (2015) 11:e1005088. doi: 10.1371/journal.pgen.1005088 25811463 PMC4374748

[5] Meissner TB Liu YJ Lee KH Li A Biswas A van Eggermond MCJA. NLRC5 cooperates with the RFX transcription factor complex to induce MHC class I gene expression. J Immunol. (2012) 188:4951–8. doi: 10.4049/jimmunol.1103160 PMC3345046 22490869

[6] Rodriguez-Rodero S González S Rodrigo L Fernández-Morera JL Martínez-Borra JM López-Vázquez A. Transcriptional regulation of MICA and MICB: a novel polymorphism in MICB promoter alters transcriptional regulation by Sp1. Eur J Immunol. (2007) 37:1938–53. doi: 10.1002/eji.200737031 17557375

[7] Chen QY Jackson N. Human CD1D gene has TATA boxless dual promoters: an SP1-binding element determines the function of the proximal promoter. J Immunol. (2004) 172:5512–21. doi: 10.4049/jimmunol.172.9.5512 15100293

[8] Chen QY Zhang T Pincus SH Wu S Ricks D Liu D. Human CD1D gene expression is regulated by LEF-1 through distal promoter regulatory elements. J Immunol. (2010) 184:5047–54. doi: 10.4049/jimmunol.0901912 20363964

[9] Groh V Bahram S Bauer S Herman A Beauchamp M Spies T. Cell stress-regulated human major histocompatibility complex class I gene expressed in gastrointestinal epithelium. Proc Natl Acad Sci U.S.A. (1996) 93:12445–50. doi: 10.1073/pnas.93.22.12445 PMC38011 8901601

[10] Narayanan GA Nellore A Tran J Worley AH Meermeier EW Karamooz E. Alternative splicing of MR1 regulates antigen presentation to MAIT cells. Sci Rep. (2020) 10:15429. doi: 10.1038/s41598-020-72394-9 32963314 PMC7508857

[11] Gobin SJ van den Elsen PJ. Transcriptional regulation of the MHC class Ib genes HLA-E, HLA-F, and HLA-G. Hum Immunol. (2000) 61:1102–7. doi: 10.1016/S0198-8859(00)00198-1 11137213

[12] Grimholt U Lukacs M. MHC class I evolution; from Northern pike to salmonids. BMC Ecol Evol. (2021) 21:3. doi: 10.1186/s12862-020-01736-y 33514321 PMC7853315

[13] Dijkstra JM Katagiri T Hosomichi K Yanagiya K Inoko H Ototake M. A third broad lineage of major histocompatibility complex (MHC) class I in teleost fish; MHC class II linkage and processed genes. Immunogenetics. (2007) 59:305–21. doi: 10.1007/s00251-007-0198-6 17318646

[14] Grimholt U Tsukamoto K Azuma T Leong J Koop BF Dijkstra JM. A comprehensive analysis of teleost MHC class I sequences. BMC Evol Biol. (2015) 15:32. doi: 10.1186/s12862-015-0309-1 25888517 PMC4364491

[15] Svenning S Gondek-Wyrozemska AT van der Wal YA Robertsen B Jensen I Jørgensen JB. Microbial danger signals control transcriptional induction of distinct MHC class I L lineage genes in atlantic salmon. Front Immunol. (2019) 10:2425. doi: 10.3389/fimmu.2019.02425 31681311 PMC6797598

[16] Grimholt U Fosse JH Sundaram AYM. Selective stimulation of duplicated atlantic salmon MHC pathway genes by interferon-gamma. Front Immunol. (2020) 11:571650. doi: 10.3389/fimmu.2020.571650 33123146 PMC7573153

[17] Gessani S Conti L Del Cornò M Belardelli B. Type I interferons as regulators of human antigen presenting cell functions. Toxins (Basel). (2014) 6:1696–723. doi: 10.3390/toxins6061696 PMC4073125 24866026

[18] Gobin SJ van Zutphen M Woltman AM van den Elsen PJ. Transactivation of classical and nonclassical HLA class I genes through the IFN-stimulated response element. J Immunol. (1999) 163:1428–34. doi: 10.4049/jimmunol.163.3.1428 10415043

[19] Jongsma MLM Guarda G Spaapen RM. The regulatory network behind MHC class I expression. Mol Immunol. (2019) 113:16–21. doi: 10.1016/j.molimm.2017.12.005 29224918

[20] Ivashkiv LB Donlin LT. Regulation of type I interferon responses. Nat Rev Immunol. (2014) 14:36–49. doi: 10.1038/nri3581 24362405 PMC4084561

[21] McNab F Mayer-Barber KSher A Wack A O'Garra A. Type I interferons in infectious disease. Nat Rev Immunol. (2015) 15:87–103. doi: 10.1038/nri3787 25614319 PMC7162685

[22] Landis ED Purcell MK Thorgaard GH Wheeler PA Hansen JD. Transcriptional profiling of MHC class I genes in rainbow trout infected with infectious hematopoietic necrosis virus. Mol Immunol. (2008) 45:1646–57. doi: 10.1016/j.molimm.2007.10.003 18187194

[23] Jorgensen SM Hetland DL McL. CP Grimholt U Gjøen T. Effect of early infectious salmon anaemia virus (ISAV) infection on expression of MHC pathway genes and type I and II interferon in Atlantic salmon (Salmo salar L.) tissues. Fish Shellfish Immunol. (2007) 23:576–88. doi: 10.1016/j.fsi.2007.01.005 17478098

[24] Dijkstra JM Yoshiura Y Kiryu I Aoyagi K Köllner B Fischer U. The promoter of the classical MHC class I locus in rainbow trout (Oncorhynchus mykiss). Fish Shellfish Immunol. (2003) 14:177–85. doi: 10.1006/fsim.2002.0431 12526881

[25] Svingerud T Solstad T Sun B Nyrud MLJ Kileng Ø Greiner-Tollersrud L. Atlantic salmon type I IFN subtypes show differences in antiviral activity and cell-dependent expression: evidence for high IFNb/IFNc-producing cells in fish lymphoid tissues. J Immunol. (2012) 189:5912–23. doi: 10.4049/jimmunol.1201188 23169587

[26] Chang CJ Jenssen I Robertsen B. Protection of Atlantic salmon against salmonid alphavirus infection by type I interferons IFNa, IFNb and IFNc. Fish Shellfish Immunol. (2016) 57:35–40. doi: 10.1016/j.fsi.2016.08.020 27530458

[27] Robertsen B. The role of type I interferons in innate and adaptive immunity against viruses in Atlantic salmon. Dev Comp Immunol. (2018) 80:41–52. doi: 10.1016/j.dci.2017.02.005 28196779

[28] Robertsen B Greiner-Tollersrud L. Atlantic salmon type I interferon genes revisited. Fish Shellfish Immunol. (2024) 151:109694. doi: 10.1016/j.fsi.2024.109694 38871143

[29] Morales-Lange B Ramírez-Cepeda F Schmitt P Guzmán F Lagos L Øverland M. Interferon gamma induces the increase of cell-surface markers (CD80/86, CD83 and MHC-II) in splenocytes from atlantic salmon. Front Immunol. (2021) 12:666356. doi: 10.3389/fimmu.2021.666356 34054836 PMC8155612

[30] Robertsen B Greiner-Tollersrud L Jorgensen LG. Analysis of the Atlantic salmon genome reveals a cluster of Mx genes that respond more strongly to IFN gamma than to type I IFN. Dev Comp Immunol. (2019) 90:80–9. doi: 10.1016/j.dci.2018.09.004 30195710

[31] Zou J Secombes CJ. Teleost fish interferons and their role in immunity. Dev Comp Immunol. (2011) 35:1376–87. doi: 10.1016/j.dci.2011.07.001 21781984

[32] Zanin N Viaris de Lesegno C Lamaze C Blouin CM. Interferon receptor trafficking and signaling: journey to the cross roads. Front Immunol. (2020) 11:615603. doi: 10.3389/fimmu.2020.615603 33552080 PMC7855707

[33] Sun B Greiner-Tollersrud L Koop BF Robertsen B. Atlantic salmon possesses two clusters of type I interferon receptor genes on different chromosomes, which allows for a larger repertoire of interferon receptors than in zebrafish and mammals. Dev Comp Immunol. (2014) 47:275–86. doi: 10.1016/j.dci.2014.08.007 25149134

[34] Aksnes I Braaen S Markussen T Åkesson CP Villoing S Rimstad E. Genetically modified attenuated salmonid alphavirus: A potential strategy for immunization of Atlantic salmon. J Fish Dis. (2021) 44:923–37. doi: 10.1111/jfd.13352 33591590

[35] Aksnes I Markussen T Braaen S Rimstad E. Mutation of N-glycosylation sites in salmonid alphavirus (SAV) envelope proteins attenuate the virus in cell culture. Viruses. (2020) 12(10):1071. doi: 10.3390/v12101071 PMC7650630 32987930

[36] Karlsen M Tingbø T Solbakk IT Evensen Ø Furevik A Aas-Eng A. Efficacy and safety of an inactivated vaccine against Salmonid alphavirus (family Togaviridae). Vaccine. (2012) 30:5688–94. doi: 10.1016/j.vaccine.2012.05.069 22691434

[37] Rodriguez Saint-Jean S Håvarstein LS Djupvik HO Ness S Endresen C. Establishment and characterization of a new cell line (SSP-9) derived from Atlantic salmon Salmo salar that expresses type I ifn. J Fish Biol. (2014) 85:1526–45. doi: 10.1111/jfb.12503 25230295

[38] Christie KE Håvarstein LS Djupvik HO Ness S Endresen C. Characterization of a new serotype of infectious pancreatic necrosis virus isolated from Atlantic salmon. Arch Virol. (1988) 103:167–77. doi: 10.1007/BF01311090 3214272

[39] Pedersen T Skjesol A Jorgensen JB. VP3, a structural protein of infectious pancreatic necrosis virus, interacts with RNA-dependent RNA polymerase VP1 and with double-stranded RNA. J Virol. (2007) 81:6652–63. doi: 10.1128/JVI.02831-06 PMC1900092 17428850

[40] Dobos P Roberts TE. The molecular biology of infectious pancreatic necrosis virus: a review. Can J Microbiol. (1983) 29:377–84. doi: 10.1139/m83-062 6303539

[41] van der Wal YA Nordli H Akandwanaho A Greiner-Tollersrud L Kool J Jørgensen JB. CRISPR-Cas- induced IRF3 and MAVS knockouts in a salmonid cell line disrupt PRR signaling and affect viral replication. Front Immunol. (2023) 14:1214912. doi: 10.3389/fimmu.2023.1214912 37588594 PMC10425769

[42] Sobhkhez M Joensen LL Greiner Tollersrud L Strandskog G Thim HL Jørgensen JB. A conserved inhibitory role of suppressor of cytokine signaling 1 (SOCS1) in salmon antiviral immunity. Dev Comp Immunol. (2017) 67:66–76. doi: 10.1016/j.dci.2016.11.001 27818171

[43] Sun B Skjæveland I Svingerud T Zou J Jørgensen J Robertsen B. Antiviral activity of salmonid gamma interferon against infectious pancreatic necrosis virus and salmonid alphavirus and its dependency on type I interferon. J Virol. (2011) 85:9188–98. doi: 10.1128/JVI.00319-11 PMC3165780 21697489

[44] Jorgensen JB Johansen A Stenersen B Sommer AI. CpG oligodeoxynucleotides and plasmid DNA stimulate Atlantic salmon (Salmo salar L.) leucocytes to produce supernatants with antiviral activity. Dev Comp Immunol. (2001) 25:313–21. doi: 10.1016/S0145-305X(00)00068-9 11246071

[45] Messeguer X Escudero R Domènec F Núñez O Martínez J Albà MM. PROMO: detection of known transcription regulatory elements using species-tailored searches. Bioinformatics. (2002) 18:333–4. doi: 10.1093/bioinformatics/18.2.333 11847087

[46] Skjesol A Aamo T Nøst Hegseth M Robertsen B Jørgensen JB. The interplay between infectious pancreatic necrosis virus (IPNV) and the IFN system: IFN signaling is inhibited by IPNV infection. Virus Res. (2009) 143:53–60. doi: 10.1016/j.virusres.2009.03.004 19463721 PMC7114382

[47] Castro R Martin SAM Bird S Lamas J Secombes CJ. Characterisation of gamma-interferon responsive promoters in fish. Mol Immunol. (2008) 45:3454–62. doi: 10.1016/j.molimm.2008.03.015 18457879

[48] Goodbourn S Didcock L Randall RE. Interferons: cell signalling, immune modulation, antiviral response and virus countermeasures. J Gen Virol. (2000) 81:2341–64. doi: 10.1099/0022-1317-81-10-2341 10993923

[49] Reya T Clevers H. Wnt signalling in stem cells and cancer. Nature. (2005) 434:843–50. doi: 10.1038/nature03319 15829953

[50] Rojas JM Alejo A Martín V Sevilla N. Viral pathogen-induced mechanisms to antagonize mammalian interferon (IFN) signaling pathway. Cell Mol Life Sci. (2021) 78:1423–44. doi: 10.1007/s00018-020-03671-z PMC7576986 33084946

[51] Fielding CA Aicheler R Stanton RJ Wang ECY Han S Seirafian S. Two novel human cytomegalovirus NK cell evasion functions target MICA for lysosomal degradation. PloS Pathog. (2014) 10:e1004058. doi: 10.1371/journal.ppat.1004058 24787765 PMC4006889

[52] Halenius A Gerke C Hengel H. Classical and non-classical MHC I molecule manipulation by human cytomegalovirus: so many targets-but how many arrows in the quiver? Cell Mol Immunol. (2015) 12:139–53. doi: 10.1038/cmi.2014.105 PMC4654289 25418469

