# Supplementary material for: Subgroup specific transcriptional regulation of salmonid non-classical MHC class I L lineage genes following viral challenges and interferon stimulations
Source: Front Immunol. 2024 Dec 20;15:1463345. doi: 10.3389/fimmu.2024.1463345 (PMC11695323; doi:10.3389/fimmu.2024.1463345)
Supplement: Supplementary file 1 [file DataSheet1.docx]

***Subgroup specific transcriptional regulation of non-classical MHC class I L linegae genes.***

***Imam et al.***

**SUPPLEMENTAL INFORMATION**

sFigure 1:

***sFig 1: Transcriptional regulation of select L lineage, Type I IFNa, IFNc and type II IFNγ genes in response to SAV3 infection in vivo.*** Pre-smolt Atlantic salmon were intra muscularly (i.m) injected with 200 μl cell culture media containing 1 × 10^2^ TCID_50_ of rSAV3. Control groups were injected with an equivalent volume of PBS and samples were collected at 2-, 4-, 6- and 8-weeks post infection (wpi). After sampling at week 8, twelve shedder fish infected with SAV3 (H20/03/2) were added to the rSAV3 injected group and samples were collected at 10 and 12-week timepoints. Log2 fold  change in gene expression of select L lineage MHC class I genes **(A, C, E, G)** and IFNa, IFNc and IFNγ (**B, D, F, H**) genes normalized to the reference gene EF1α, in different tissues at various times post challenge (*n* ϵ 3-6), relative to the control groups from the same time point (*n* ϵ 6) is shown. Each dot represents an individual fish, asterisks indicate the strength of significance among the different time points as indicated, **p* < 0.05, ***p* < 0.01, ****p* = 0.0001, *****p* < 0.0001.


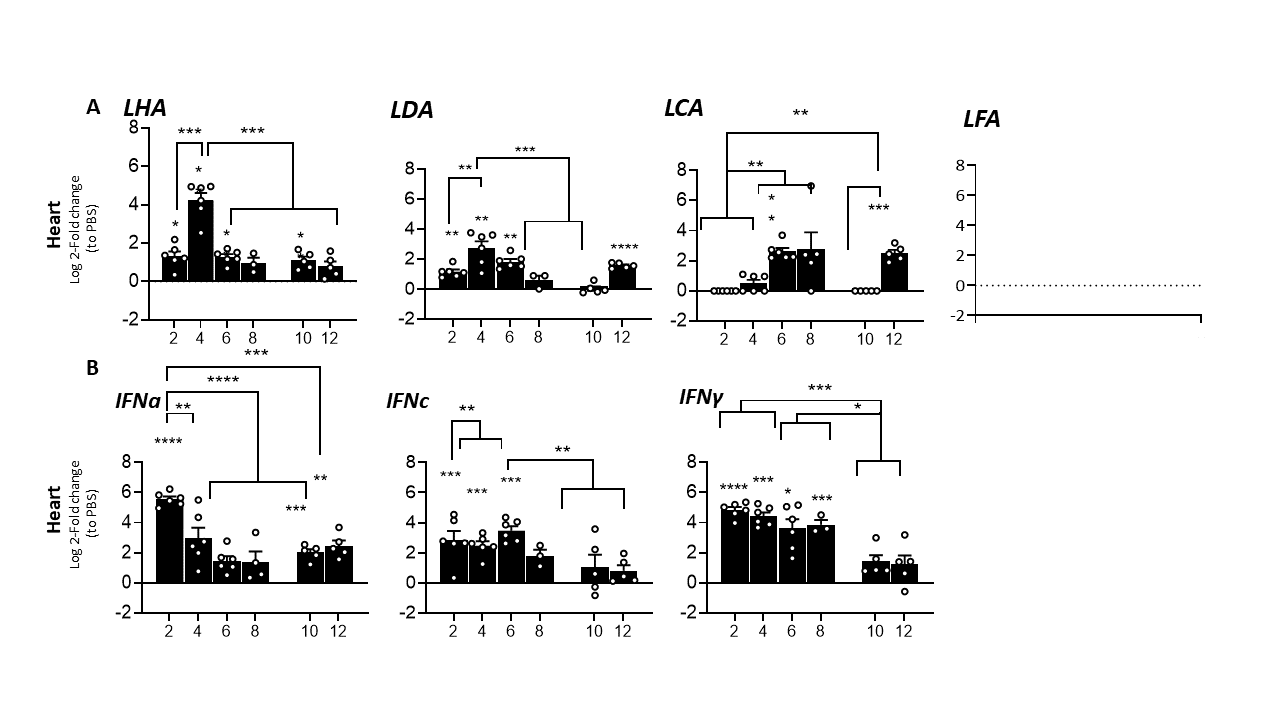

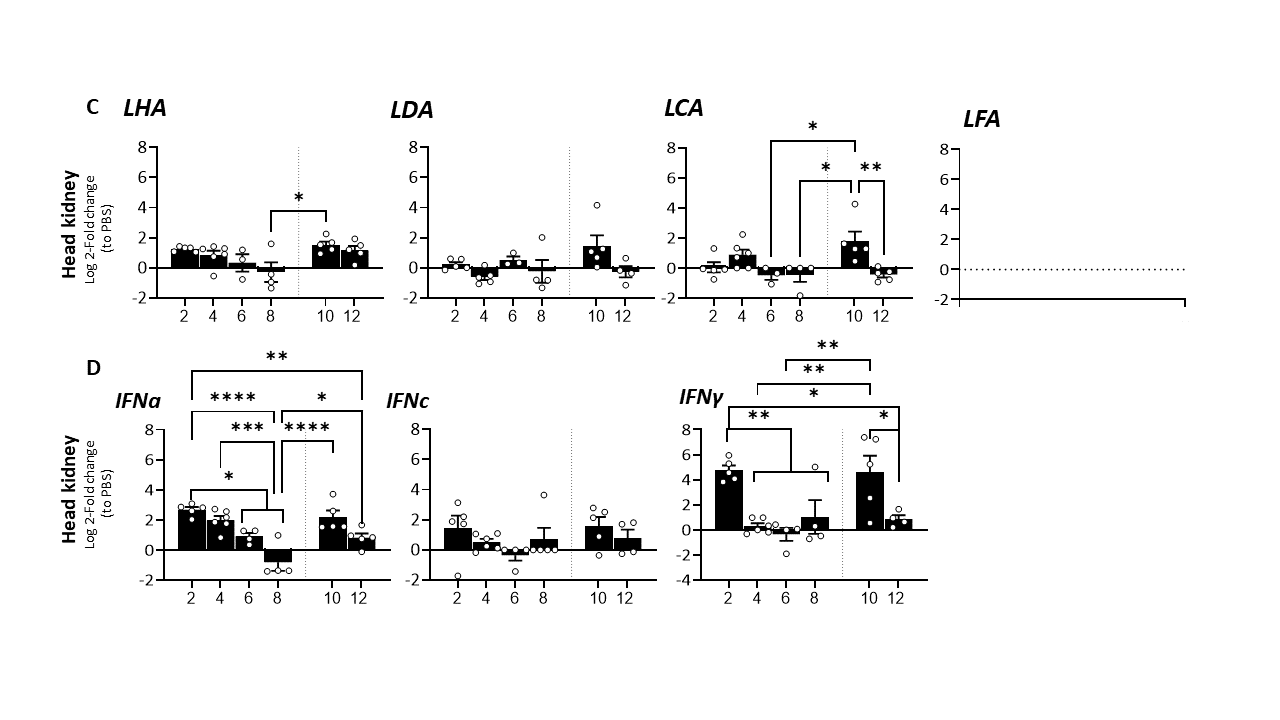


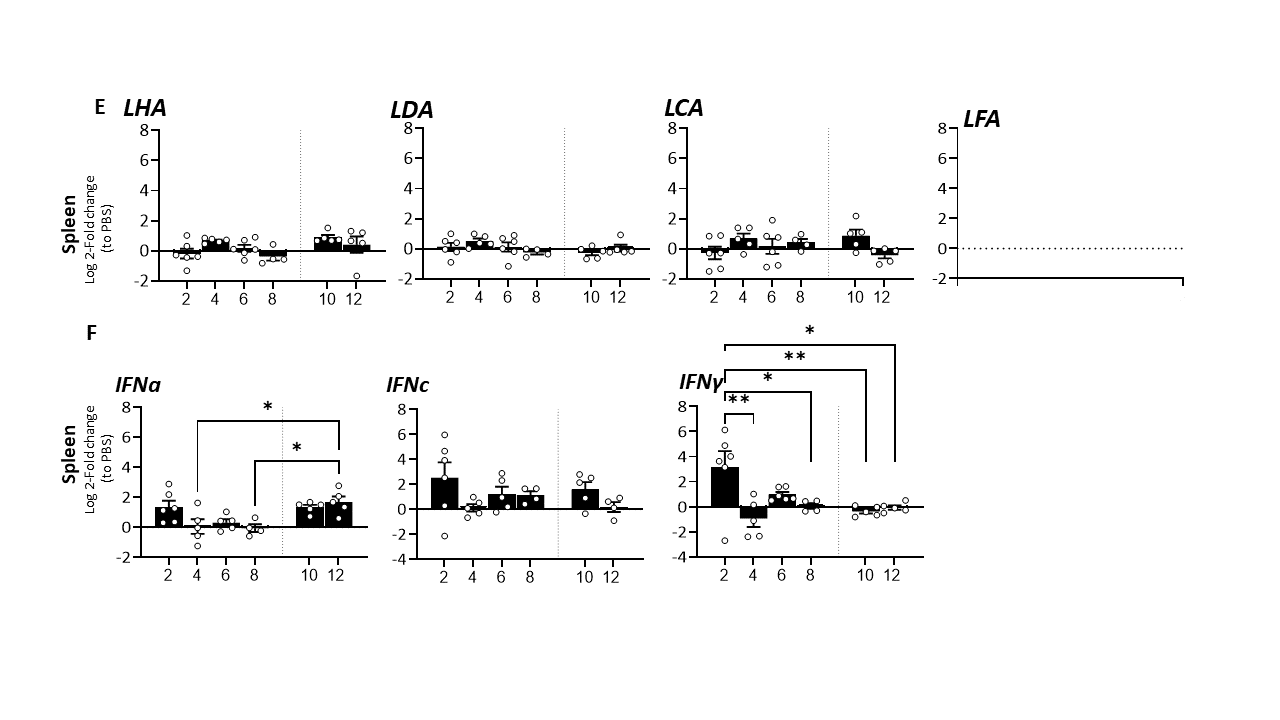

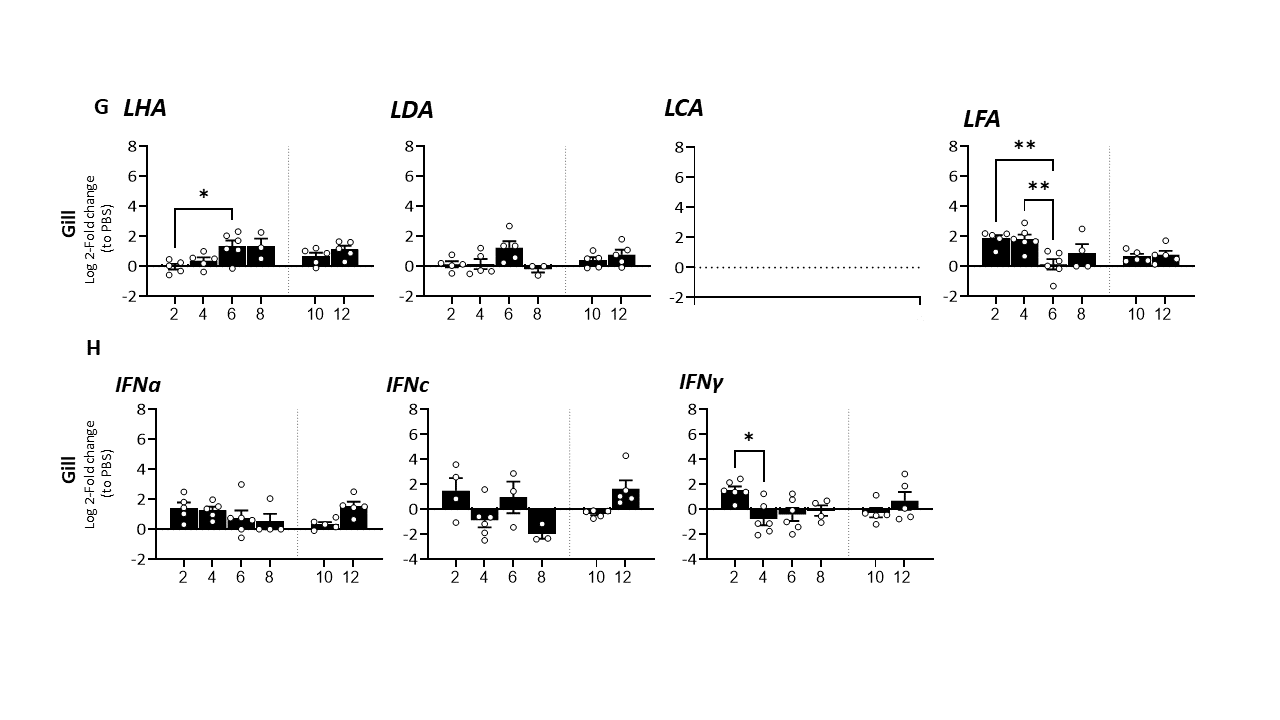


***sFig 2: Transcriptional profiling of select genes in SAV3 infected SSP-9 cells.*** Relative gene expression of **(A)** IFNa, **(B)** Mx1/2, **(C)** Mx8 and **(D)** Onts-LDA measure 1-10 days post SAV3 infection in SSP-9 cells (MOI =1 and MOI = 5). Bars represent mean ± SE (n = 3/time point with individual results shown as white dots) expressed as fold induction compared to non-infected cells at each time point, asterisks indicate the strength of significance among the different time points as indicated, **p* < 0.05, ***p* < 0.01, ****p* = 0.0001, *****p* < 0.0001. All samples were analyzed with RT-qPCR and normalized against EF1α as reference gene.

**A B C D**


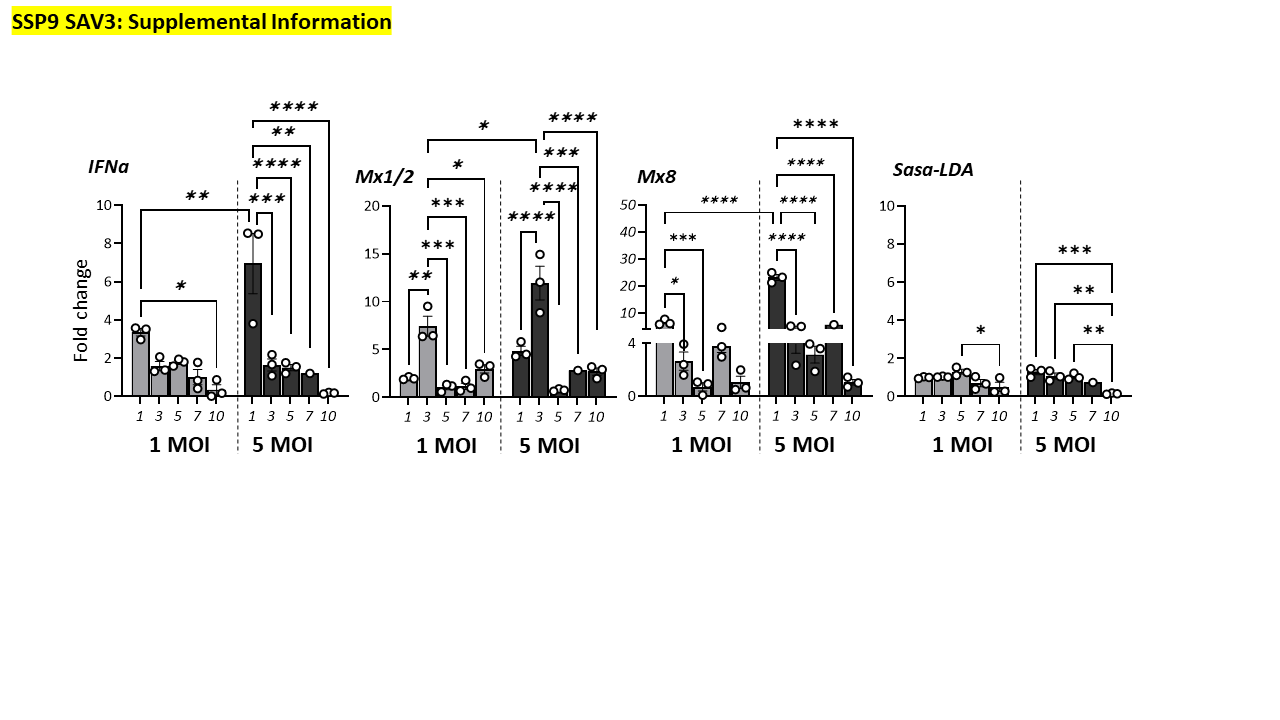


***sFigure 3: Comparison of LIA and LGA1 sequences in Atlantic salmon and chinook salmon.*** Multiple amino acid alignments (CLUSTALW) of deduced amino acid sequences between **(A)** Sasa-LIA and Onts-LIA and **(B)** Sasa-LGA1 and Onts-LGA1. The sequences are divided into leader, G-alpha 1, G-alpha 2, C-like, transmembrane (TM) and cytoplasmic tails (CYT) as indicated in the figure, identical residues are highlighted in yellow and stop codons are depicted with a *. Sasa-LIA and Sasa-LGA1 sequences were obtained from the current Atlantic salmon reference genome (GCF_905237065.1, Ssal_v3.1) and Onts-LIA and Onts-LGA1 sequences were obtained from the current Chinook salmon reference genome (GCF_018296145.1, Otsh_V2.0)

**A**


**B**

***sFig 4: Transcriptional profiling of select genes in SAV3 infected CHSE-214 cells.*** Relative gene expression of (A) Onts-LDA, (B) Onts-LEA, (C) IFNc (D) IFNγ, (E) Mx1/2, (F) Mx8 and (G) CXCL10 measure 1-12 days post SAV3 infection in CHSE-214 cells (MOI =1 and MOI = 5). Bars represent mean ± SE (n = 4/time point with individual results shown as white dots) expressed as fold induction compared to non-infected cells at each time point. The red line intersecting the y-axis at 1 represents the unstimulated control that the fold change of the treatments is in relation to. Significant fold changes (p < 0.05) are indicated by asterisks and indicate the strength of significance among the different time points as indicated, **p* < 0.05, ***p* < 0.01, ****p* = 0.0001, *****p* < 0.0001. All samples were analyzed with RT-qPCR and normalized against EF1α as reference gene.


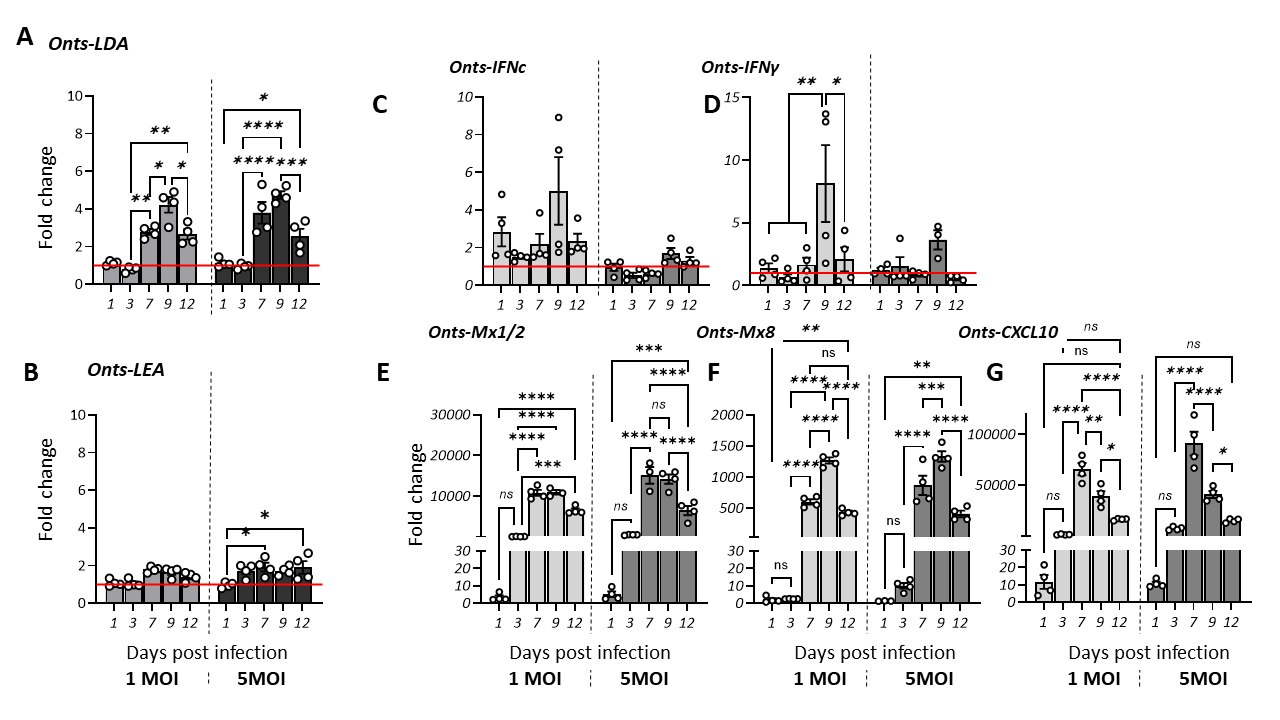


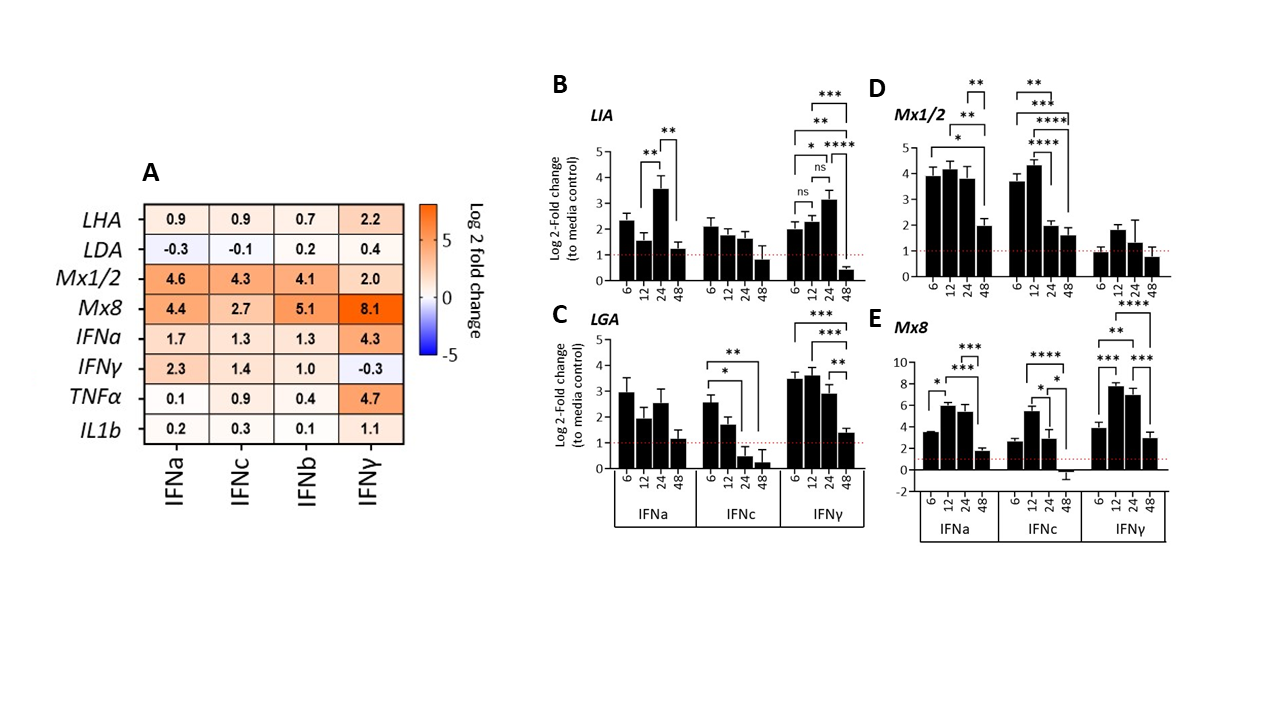
***sFig 5: Transcriptional analysis of HKLs stimulated with Type I IFNa1, INFb and IFNc compared to type II IFNγ****. HKLs were stimulated with 500U of either rIFNa1, rIFNc or rIFNb or10ng/ml rIFNg and analysed. Gene expression data at each time point were normalized against the reference gene EF1αB and log2- fold changes were calculated using the unstimulated sample (media alone) collected at the same time point. The data represent values from [n ϵ (5;8)] individuals.* ***(A)*** *The heat map illustrates average expression ratios as log2 fold-change values of Sasa-LHA, Sasa-LDA, Mx1/2, Mx8, IFNa, IFNy, TNFa and IL1b in HKLs stimulated for 24 h compared to unstimulated cells.* ***(B-E)*** *Gene expression expressed as log2-fold change in Sasa-LIA, Sasa-LGA1,Mx1/2 and Mx8 expression compared to control cells collected at the corresponding timepoint in HKL stimulated with 500U of rIFNa1 or rIFNc compared to 10ng/ml rIFNy, and collected at 6-, 12-, 24- and 48-hours post stimulation. The data represent values from [n ϵ (4;8)] individuals.The line intersecting the y-axis at 1 represents the unstimulated control that the fold change of the treatments is in relation to. Asterisks indicates the strength of significance: *p < 0.05, **p < 0.01, ***p < 0.001, and ****p < 0.0001. among the indicted bars. The data presented is representative of two separate experiments.*

***sFig6. Effect of PAN JAK inhibitor on basal expression of Sasa-LIA, Sasa-LGA1 and IFNa gene expression in SSP-9 cells.*** Relative gene expression of (A) Sasa-LGA1, (B) Sasa-LIA and (C) IFNa in SSP-9 cells incubated for 24h in the presence (striped bars) or absence (white bars) of 15nM JAK 1 inhibitor(In SolutionTM JAK Inhibitor; Calbiochem).Gene expression data at each time point were normalized against the reference gene EF1αB. The data represent values from [n ϵ (3)] technical replicateswith each dot indicating a replicate and the data presented is representative of three separate experiments. P values are indicated above the respective bars.

**A B C**


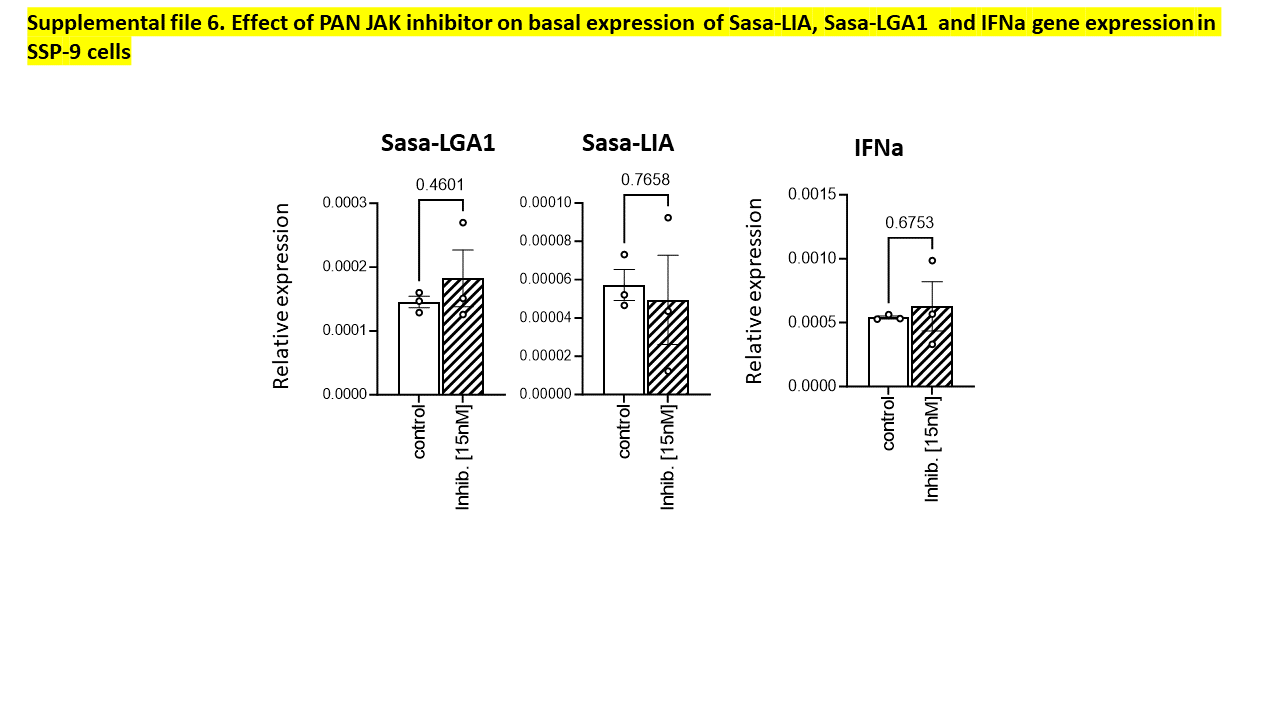


***sFig 7: Pharmacological inhibition of the JAK/STAT pathway reduces rIFNa1, rIFNc and rIFNγ-induced L lineage expression in HKLs.*** Gene expression of **(A)** Sasa-LIA, **(B)** Sasa-LGA1, **(C)** Mx8 and **(D)** Mx1/2, in HKLs stimulated for 16 hours with 500U IFNa, 500U IFNc or 10ng IFNγ, in the presence (striped bars) or absence (solid bars) of 15nM JAK 1 inhibitor (In SolutionTM JAK Inhibitor; Calbiochem). Gene expression data at each time point were normalized against the reference gene EF1αB and fold changes were calculated using cells incubated with media containing the inhibitor as reference. Each dot represents an individual fish. The data presented is representative of three separate experiments. Asterisks above each bar indicate significant upregulation compared to control cells while asterisks above two indicated bars indicate significant reduction in upregulation with inhibitor compared to cells stimulated in absence of the inhibitor.


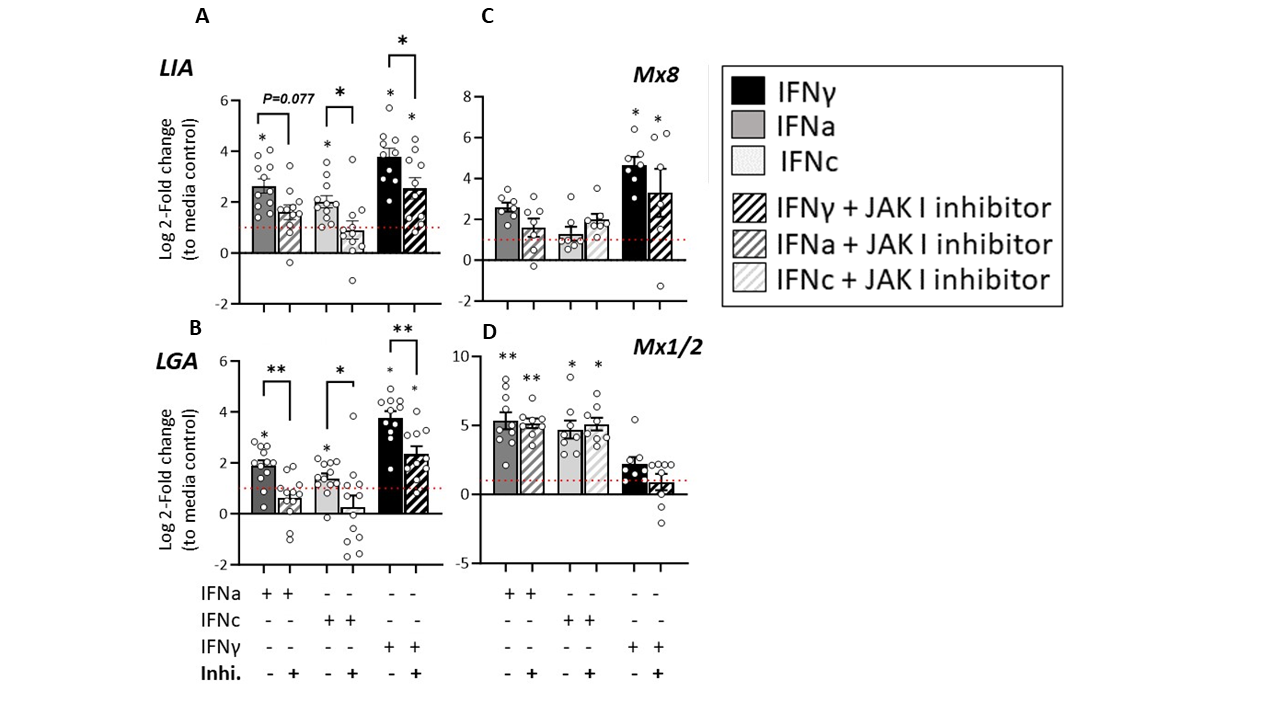


***sFig 8: Sequence analysisis of the promoter regions of Sasa-LIA and Sasa-LGA1 genes.*** **(A)** Schematic represntation of the 5′ flanking sequence (-1 to -2000bp) of Sasa-LIA and Sasa-LGA1. ISRE elements are indicated in green, Gas-like elements are indicted in blue and STAT/IRF core elements are indictaed in black and grey repsectively. The proximal promoter region seqeunce (-1 to -500bp) of **(B)** Sasa-LIA and **(C)** Sasa-LGA1 is shown with putative binding sites indicated.


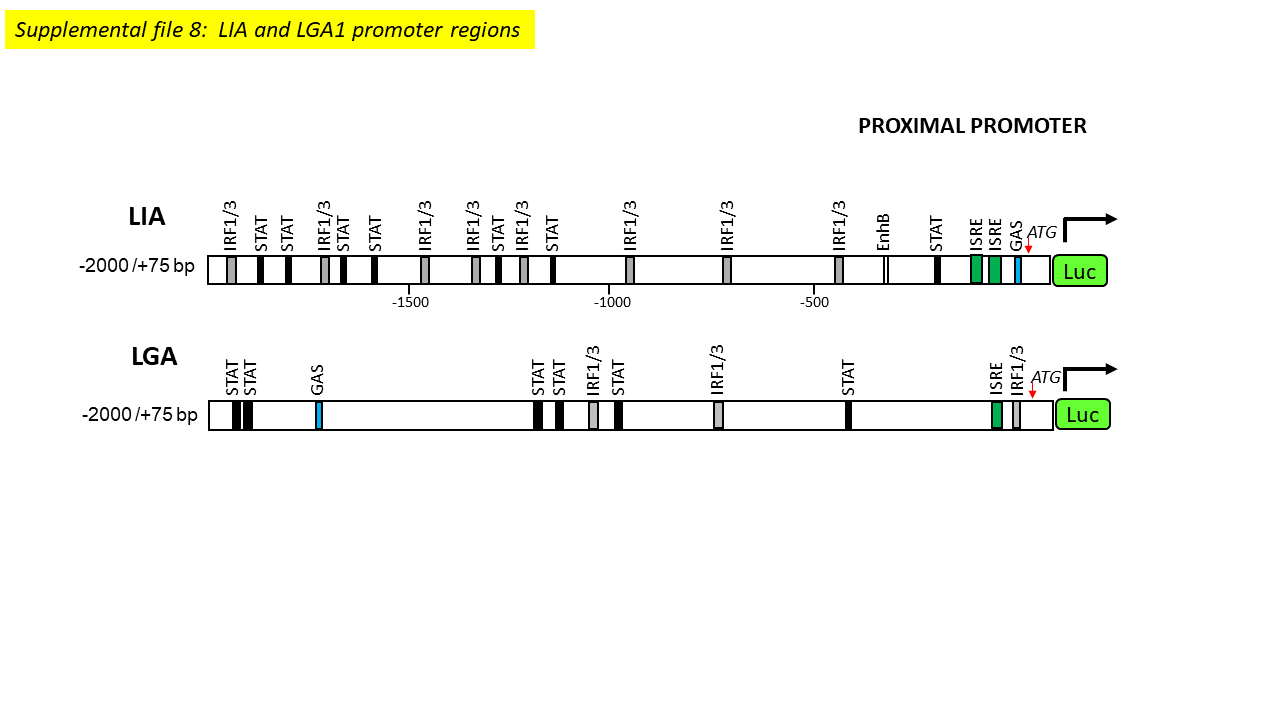
**A**

**B**

**C**
